# Phage-based delivery systems: engineering, applications, and challenges in nanomedicines

**DOI:** 10.1186/s12951-024-02576-4

**Published:** 2024-06-25

**Authors:** Hui Wang, Ying Yang, Yan Xu, Yi Chen, Wenjie Zhang, Tianqing Liu, Gang Chen, Kaikai Wang

**Affiliations:** 1 School of Pharmacy, Nantong University, Nantong, 226001 China; 2 Qingdao Central Hospital, University of Health and Rehabilitation Sciences (Qingdao Central Medical Group), Qingdao, 266024 China; 3 School of Rehabilitation Sciences and Engineering, University of Health and Rehabilitation Sciences, Qingdao, 266024 China; 4 NICM Health Research Institute, Western Sydney University, Sydney, NSW 2145 Australia

**Keywords:** Phage, Drug and gene delivery, Targeting, Phage engineering, Nanocarriers

## Abstract

Bacteriophages (phages) represent a unique category of viruses with a remarkable ability to selectively infect host bacteria, characterized by their assembly from proteins and nucleic acids. Leveraging their exceptional biological properties and modifiable characteristics, phages emerge as innovative, safe, and efficient delivery vectors. The potential drawbacks associated with conventional nanocarriers in the realms of drug and gene delivery include a lack of cell-specific targeting, cytotoxicity, and diminished in vivo transfection efficiency. In contrast, engineered phages, when employed as cargo delivery vectors, hold the promise to surmount these limitations and attain enhanced delivery efficacy. This review comprehensively outlines current strategies for the engineering of phages, delineates the principal types of phages utilized as nanocarriers in drug and gene delivery, and explores the application of phage-based delivery systems in disease therapy. Additionally, an incisive analysis is provided, critically examining the challenges confronted by phage-based delivery systems within the domain of nanotechnology. The primary objective of this article is to furnish a theoretical reference that contributes to the reasoned design and development of potent phage-based delivery systems.

**Graphical abstract:**

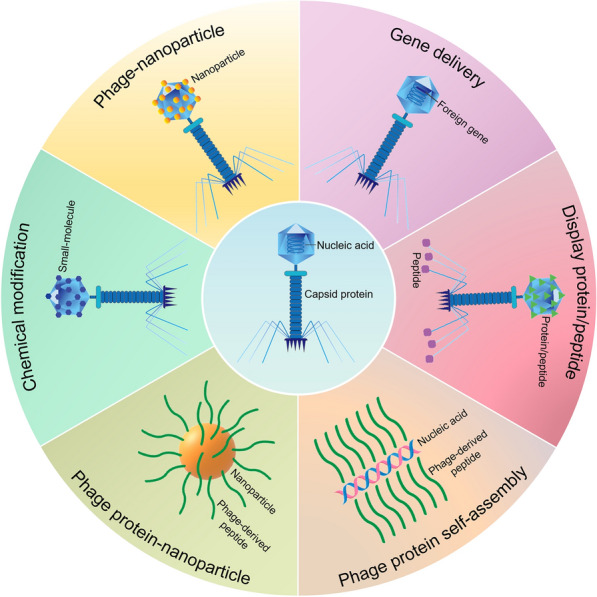

## Introduction

The emergence of nanotechnology opened up a new path for precision medicine, a large number of nanocarriers have been exploited and demonstrated good delivery performance, including liposomes, polymeric nanoparticles, micelles, viruses and stem cells [1]. Generally, an ideal delivery vector should have some special properties, such as good biocompatibility, targeting specificity, low toxicity, and high uptake efficiency. However, each of these existing nanocarriers has its advantages and disadvantages in terms of general requirements for therapeutic applications [2]. For example, liposomes are widely used as suitable nanocarriers for multiple drug deliveries due to their structural versatility, biocompatibility, biodegradability, non-toxicity, and non-immunogenicity nature [3, 4]. However, the instability of liposomes in biological media is prone to drug leakage, as well as their sensitivity to various external parameters, such as temperature and osmotic pressure [5]. While some organic nanoparticles are commonly used as carriers taking advantages of their targeting and delivery profile [6], some metal-based inorganic nanoparticles show unique optical properties, potential magnetic and catalytic properties. However, the circulation issue and potential toxicity of certain inorganic nanoparticles in vivo limit their application in delivery [7–9]. While protein nanoparticles have great biocompatibility, biodegradability and low immunogenicity, there are still some problems that cannot be ignored, such as protein instability, nonuniform particle size during preparation, and unclear transport mechanism [10, 11]. In recent years, the burgeoning field of phage research has unveiled promising applications that address some of the limitations encountered with conventional nanocarriers.

Phages, ubiquitous viruses found in nature, were independently discovered by Frederick Twort and Félix d’Hérelle in 1915 and 1917 [12]. Comprising primarily nucleic acids and capsid proteins, phages can be conceptualized as protein nanoparticles that encapsulate their genetic materials. Phages exist in a variety of shapes and sizes, including icosahedral and filamentous phages [13]. Phages undergo distinct life cycles, categorized as lytic and temperate phages. After infecting bacteria, lytic phages (such as T4 phage) use the transcription and translation system of the host bacteria to synthesize their own proteins, assemble new progeny phages, and then cleavage host cells to release progeny phages, resulting in the death of host cells. While temperate phages (such as M13 phage) do not cause the death of the host bacteria after infecting the host bacteria. Instead, they integrate their own genetic materials into the genome of the host bacteria, where they replicate [14, 15].

Phages have high biosafety profiles because of their specific targeting of bacteria without infecting mammalian cells [16]. They are also biocompatible and maintain relatively stable biological activity across a broad spectrum of environmental conditions, including high temperatures, varied pH ranges, and in the presence of nucleases and proteases [17]. In the 1980s, Smith [18] inserted the DNA of a foreign peptide into the pIII capsid protein gene of filamentous phage f1 for the first time. The polypeptide encoded by the foreign gene was displayed on the surface of the phage in the form of fusion protein, resulting in the establishment of phage display technology. This breakthrough marked a significant milestone in new nanomaterial development and spurred the emergence of phage-based delivery systems [19]. The abundance of coat proteins on the phage surface facilitates the display of various peptides or proteins, which can be used for site-specific targeting or as active molecules. Moreover, genetic engineering enables tailored modifications for targeted designs [20]. In comparison to conventional nanocarriers, phages offer several advantages, including ease of manipulation, versatility in peptide or antibody display, technical simplicity in preparation and purification, and cost-effectiveness in large-scale production [21]. The progression of phage research has paved new avenues for drug and gene delivery applications. Phages themselves or in combination with other nanocarriers serve as versatile vectors for drug and gene transport. Drugs can be chemically conjugated to the phage surface, while genes can be inserted into the phage genome through genetic engineering. Traditional nanocarriers can also be assembled with phage through physical adsorption, chemical bonding and other means to obtain new composite delivery vectors [22]. In addition, phage-derived peptides or proteins can be directly incorporated into nanomaterials or self-assembled to facilitate drug and gene delivery [1].

**Fig. 1:**
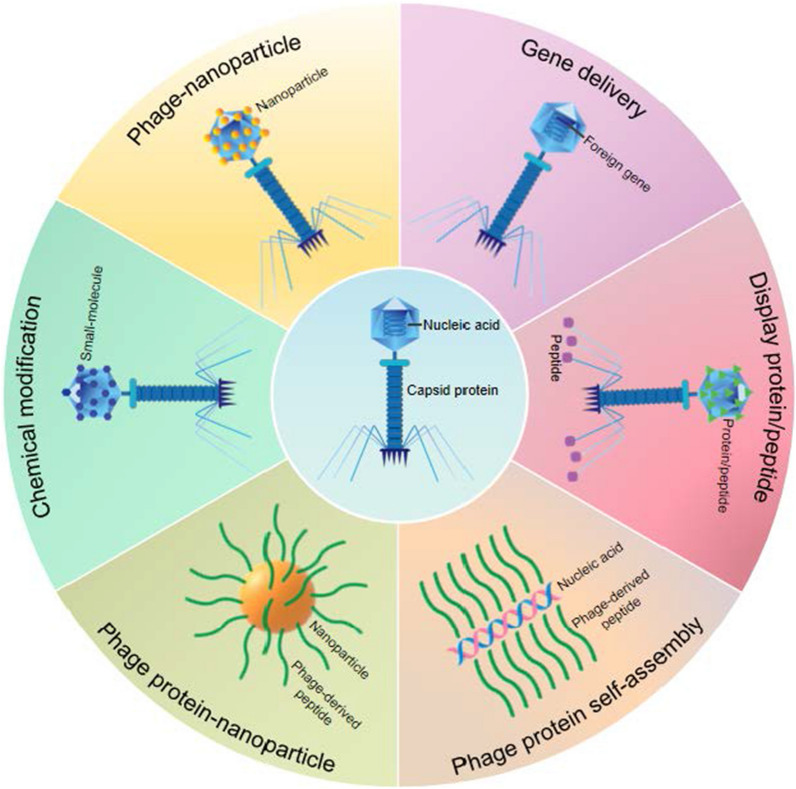
Overview of designing various phage-based delivery systems for drug and gene delivery

In this review, we present the latest research on phages as delivery vehicles (Fig. 1). We focus on the current phage engineering modification strategies and the main types of phages used for drug and gene delivery. The applications of these delivery strategies in disease treatment are discussed. In addition, we summarize the common modification techniques of specific functional groups on phage coat proteins and the modification strategies of phages as delivery carriers for disease treatment. Finally, we provide prospects on the challenges and opportunities of phages as delivery systems, laying an important foundation for its further application and research.

## Safety

Phages have been used to treat bacterial diseases since the beginning of their development and have a history of more than 100 years. In 1917, Félix d’Hérelle announced that he had isolated a microorganism resistant to Shigella, which he called these antagonists "phages" [23]. However, interest in phage therapy waned after the discovery of penicillin by Alexander Fleming in 1928, leading to a decline in its study and application, while phage therapy persisted in Eastern Europe and the former Soviet Union [22]. The widespread use of antibiotics overshadowed phage therapy until the emergence of antibiotic-resistant bacterial strains, which posed significant challenges in infection management. This resurgence, coupled with a growing understanding of infectious diseases and the demand for sustainable antimicrobial solutions, has renewed interest in phage therapy worldwide in order to combat antibiotic-resistant bacterial infections [24, 25].

Although the excellent biological characteristics and therapeutic potentials of phage therapy have been demonstrated, the safety concerns have hindered their widespread adoption in the clinical settings. One main safety issue of phages revolves around the presence of the residual endotoxin in phage preparations. Endotoxins are lipopolysaccharides (LPS) found in Gram-negative bacteria polysaccharide cell walls and released during bacterial division or death. Endotoxins are highly immunogenic, which can increase the body temperature, reduce the number of blood white blood cells, and even shock. Therefore, US FDA guidelines stipulate that the level of LPS in intravenously administered biologic drugs must be less than 5 EU per 1 kg of body weight [26–28]. Improvements in endotoxin removal processes are necessary to meet these stringent safety standards and facilitate the standardized use of phage therapies. Another challenge lies in the production of anti-phage antibodies, which can limit the therapeutic efficacy of phage preparations. In mammalian hosts, immunoglobulin M (IgM) and immunoglobulin G (IgG) antibodies in serum can reduce or inhibit phage activity, respectively. The host immune response to bacterial infection may lead to the rapid clearance of phages from the system, compromising their therapeutic effectiveness [29].

Phage can horizontally transfer genetic materials between host bacteria, especially the possibility of gene transfer encoding virulence or antibiotic resistance [30]. Phages integrate the toxin gene into the host bacteria through the process of lysogeny. Therapeutic use of phages with virulence genes in their genomes will transfer pathogenic properties to bacteria in the normal human biota, causing potential health problems for humans [31]. In addition, phages are composed of proteins and nucleic acids, which can interact with the human immune system. After entering the body, the macromolecular protein components will produce immunogenicity and may cause allergic reactions. Although phages have been reported to elicit only mild immune responses [24], constant attention is still needed. The points mentioned above are common problems encountered in the preparation of phage preparations, and other possible risks related to phage also need to be considered. Phage has the potential to threaten symbiotic bacteria, especially through the use of broad-spectrum phage preparations, which is enough to interfere with the resident microbiota, thus indirectly affecting eukaryotic organisms. Phages may evolve during manufacture or use, for example, lysed phages may be converted to lysogenic forms, thereby losing the ability to lysate bacteria [32].

Even though phage therapy has made outstanding achievements in the field of solving bacterial tolerance, overcoming these limitations is crucial for realizing the full therapeutic potential of phage-based interventions and advancing their clinical application. When screening phages, it is necessary to identify phages that exhibit excellent antimicrobial virulence, cause little harm to patients, and have the ability to reach the target bacteria in situ [33]. Ideally, phage preparations need to be characterized through whole genome sequencing to exclude virulence factors, toxin genes, and lysogenic genes. Highly purified phage preparations will be reduce allergic or toxic effects [31]. Currently, while there have been successful cases, phage preparations are costly and complex in process with limited capacity for mass production. With the continuous development of technologies, the large-scale clinical use will gradually become possible.

## Influence of phages on mammalian cells

Phages are specific viruses that parasitize bacteria by infecting and replicating [34]. Unlike eukaryotic viruses, phages cannot infect or replicate within mammalian cells. The restrictions on infection and production attributed to prokaryotic hosts protect the human host from inadvertent phage infection. As the increasing use of phages on patients clinically, the potential impact of phage-mammalian interactions is of great concern [35]. Mammalian cells can phagocytize phages through a variety of mechanisms, such as macropinocytosis, clathrin-mediated endocytosis, or caveolae-mediated endocytosis, resulting in the internalization and accumulation of phages [36]. Phages have also been shown to bind to specific mammalian cell receptors, triggering receptor-mediated endocytosis. For example, lambda phage coated with poly clonal antiserum against a capsid protein can enter kidney fibroblasts expressing the receptor FcγRI in vitro via antibody-dependent receptor endocytosis [37]. Phages can also use receptor molecular mimicry to trigger endocytosis into neuroblastoma cells by interacting with cell surface polysialic acid, which shares structural similarity with the bacterial phage receptor [38]. Among them, the main mechanism of phage entry into mammalian cells is non-specific internalization through macropinocytosis [39].

Fortunately, since the discovery of phages, phages, even high titers of phages, have demonstrated excellent biosafety profiles in humans [16]. Phages are internalized through endocytosis and processed by antigen-presenting cells, and have the ability to induce humoral and cellular immunity, making them well suited as effective adjuvants or nanocarriers for low-immunogenic vaccines [40]. More recently, Bichet et al. [39] showed that phage internalization induced broad cellular signaling cascades to promote the growth and survival of mammalian cells. Mammalian cells used internalizing phages as resources to enhance proliferation and metabolism. This study demonstrated that mammalian cells could gain benefits by interacting with phages.

The interactions between phages and mammalian cells can exhibit a breadth and diversity of effects. Nevertheless, understanding of the impact of these phages on mammalian cells and immune processes remains limited. Although existing research has offered valuable insights into the mechanisms and potential implications of phage-mammalian interactions in health and disease, numerous questions remain unanswered. There is a pressing need for further investigation into potential symbiotic interactions between phages and mammalian cells, as well as the broader effects that these interactions may exert in various contexts [35]. Future research endeavors should focus on elucidating the intricacies of phage-mammalian interactions and their implications for human health, paving the way for the development of novel therapeutic strategies and interventions.

## Current engineering strategies based on phage

### Phage direct delivery system

Phages themselves or combined with nanocarriers can be used as carriers for drug and gene delivery. Nanomaterials and therapeutic drugs, can be combined with capsid proteins through physical interactions or chemical modifications. Genes can be inserted into the phage genome by genetic engineering. In this section, we will introduce the current modification strategies for engineering phages as delivery vehicles.

#### Non-covalent modified phages as delivery vehicles

Phage has huge surface loading capacity and flexible genetic engineering properties. Nanocarriers are assembled with phages through physical adsorption, covalent binding, and other methods to obtain new composite carriers. However, the efficiency of covalent conjugation may vary depending on the functional moieties and coupling agents in the complex, as well as multi-step chemical reactions. Furthermore, specific biomolecules can easily change conformation and lose their activity during this modification [41]. Physical absorption, operating through the electrostatic interaction, van der Waals forces, hydrogen bonding, or hydrophobic interactions, often allows additional ligands to maintain conformational stability [42]. The non-covalent binding of biomolecules to nanocarriers can maintain their biological activity largely unchanged and achieve reversible release. This simple and unique strategy has also gained more and more attention in recent years.

Electrostatic assembly, a strategy for self-assembly using the charge difference between phage and nanocarriers to generate electrostatic interactions. Filamentous phages, such as M13 phage, have a unique rod-like structure with a negatively charged surface, which makes it easier for them to adsorb positively charged substances [40]. Electrostatic interactions have been widely used to prepare nanoscale drugs and gene carriers. Positively charged substances such as cationic polymers [40, 43], silver nanoparticles [44], and liposomes [45] have been used for electrostatic assembly with phages, expanding the application of phage delivery systems in the field of nanomedicine. M13 phage and polyethyleneimine (PEI) were used to prepare phage-based vaccine platforms by simple mixing. M13 phage was encapsulated with PEI driven by electrostatic force and negatively charged antigens can be successfully adsorbed by electrostatic interactions with high efficiency and stability [40]. Compared to the currently more popular strategy of using phage display technique to deliver antigens for the preparation of phage-based vaccines, the presence of displayed molecules is vulnerable to various factors, such as sequence length and antigen conformation [46]. This modification method is expected to overcome the limitation of antigen delivery. Ligand-directed phages, such as RGD4C-phage, play an important role in facilitating gene delivery, but there is still room for improvement. For example, lysosomal escape is also an important factor to consider when designing gene delivery vehicles. The electrostatic assembly of RGD4C-phage and cationic polymer can retain the targeting and specificity of gene delivery, while improving the efficiency of gene delivery [47]. Additionally, M13 phage and silver nanoparticles can play a synergistic effect through the simple combination of electrostatic assembly, relying on the accurate targeting characteristics of phage and the ability of inorganic nanomaterials to assist in killing bacteria to optimize the defects of traditional antibiotic treatment. This system can achieve the purpose of suppressing colorectal cancer (CRC) by precisely regulating the gut microbiota to reconstitute the tumor immune microenvironment [48, 49]. The combination of M13 phage and liposome, a traditional drug delivery carrier, improves the problem that liposome is unstable in biological media and easily causes drug leakage. The formed phage-liposome complex also helps to deliver the drug-loaded liposome to specific targets [45]. The above research indicated the diverse application of electrostatic assembly of phages and nanomaterials in nanomedicine, and this modification strategy was fruitful. Importantly, the safety issue of cationic polymers still cannot be ignored. The current development strategy for cationic polymers to address this issue is modification, such as PEGylation or fluorination [50].

For some nanomaterials that cannot bind to phages through electrostatic interactions, covalent modifications are usually performed on the viral capsid to connect interesting nanomaterials. For example, introducing thiol groups to modify phage capsids connects gold materials by forming gold-sulfur bonds [51]. In fact, nanomaterials may also be connected to phages through peptide-based specific interactions. The research team proposed a method of assembling AuNP on the surface of T7 phage. The AuNP was bound to the recombinant phage through specific interaction with the gold-binding peptide motif displayed on the phage. This physical adsorption process is essentially mediated by a combination of multiple interactions, including lattice matching, intermolecular interactions within peptides, and solvent surface interactions [52]. It was reported that glutamate has a good affinity for metal cations [53]. On this basis, Dong et al. [54] proposed that four repetitive glutamate sequences were expressed at the N-terminal of the pVIII protein of M13 phage, and the photothermal palladium nanoparticles (PdNPs) were successfully bound by affinity interaction. Therefore, based on the peptide-based specific interaction, appropriate affinity peptides displayed by phages can be assembled with different types of nanomaterials for a wide range of applications. Furthermore, phages and biomaterials can also co-assemble for the delivery of drug molecules. According to previous reports, the main cause of the assembly of viruses and polymers into core–shell nanoparticles is due to the fact that viral particles are hydrophilic and polymers, such as P4VP, are hydrophobic, and interfacial forces can force viral particles to completely cover the surface of polymers, thus self-assembling into spherical nanospheres. This process is similar to using viral particles to form Pickering emulsion to stabilize oil droplets in water [55]. Suthiwangcharoen et al. [56] exploited the controlled assembly of non-covalent interactions between viruses and polymers to generate core–shell nanoassemblies. Through hydrophobic interactions, drugs can be encapsulated in the hydrophobic core of micelles composed of PCL–P2VP. At the same time, based on the assembly principle of Pickering emulsion method, M13 phage and block copolymer PCL-P2VP constructed a nanoscale delivery system together and successfully encapsulating drug molecules.

In conclusion, electrostatic assembly using surface charge differences to functionalize phages is a simple and unique strategy, unlike surface chemical covalent modifications that easily interfere with the phage tail structure [57]. But it may lack persistence [58]. Nanomaterials can also bind to phage through specific peptide-based interactions or hydrophobic interactions. These non-covalent modification methods do not interfere with the specificity of the phage itself, providing novel strategies for preparing nano-drug delivery carriers.

#### Covalent modified phages as delivery vehicles

Although molecular display plays a dominant role in the conventional application of phages, non-genetic manipulation on the surface of the virus can also improve the original function of the virus, thus expanding its scope of application [59]. As a good drug delivery carrier, the phage has a large number of sites that can be chemically modified. The amino acids in the capsid protein provide a variety of reactive functional groups that can be used for bioconjugation. Chemical groups can be used as linkers to modify drugs to overcome the problems of drug therapy. The reactivity of these functional groups depends on steric accessibility, ionization state, and solvent conditions [60]. Chemical modification of phages provides, in part, a wider variety of functionalities to phages, and capsid proteins can be functionalized in various chemical reactions without loss of particle integrity [61]. Furthermore, rapid, site-specific and hydrolysis stable modification of biomolecules is crucial for their application. Therefore, it is very meaningful to conduct detailed chemical modification studies on phages. In view of this, we summarize some research methods for modifying specific functional groups on phage coat proteins to gain a comprehensive understanding of their properties (Table 1).

**Table 1:** Selected reports on common modification techniques for specific functional groups on phage coat proteins

Reactive group	Reactive compound	Reaction product	Chemical reaction	Refs.
Amine –NH_2_	NHS Ester		Amidation reaction	[86]
	TFP Ester		Amidation reaction	[87]
	Isothiocyanate		Nucleophilic addition	[17]
N-terminal amine	PLP + Oxyamino		Transamination and oximation	[69]
Carboxylate –COOH	EDC + Amine		Amidation reaction	[71]
Thiol –SH	Maleimide		Alkylation	[74]
	Iodoacetamido		Alkylation	[88]
	Tris(bromomethyl)benzene		Benzylation	[89]
Phenol	Diazonium		Diazotization	[77]
β-amino alcohol	NaIO_4_ + Oxyamino		Oxidation and oximation	[90]
Azide	Alkyne		Click chemistry reaction	[91]

Amino groups are present at the N-terminus of proteins and on lysine side chains, and the formation of amide bond between the amino group of the coat protein and the acylation reagent is the most widely used bioconjugation strategy. N-terminal amines and lysine residues have been used in acylation sites to combine final functional groups or chemical junctions for further chemical modification [62]. The pH can promote the reaction toward the N-terminus α-amino group (pKa ~ 8) or the ε-amino group of lysine (pKa ~ 10), although the N-terminal amino group has higher solvent accessibility and lower pKa are preferentially targeted, but often still results in mixed modifications due to lack of specificity [58]. NHS esters are widely used because they are highly efficient and do not require harsh reaction conditions. This reaction proceeds in the ideal pH range of 7.0–9.2 and forms a stable amide bond [63]. NHS esters have been used to modify phages from various families, including M13, T4, and MS2. NHS ester has been used to react with the amine groups on M13 phage surface to generate thiol-modified M13 phages for coupling with Ag coated Au nanoparticles (AuNPs@Ag) [64]. Besides, NHS esters in homo- and hetero-bifunctional reagents have been used to covalently combine T4 phages with gold sensors and cross-link MS2 capsids with cell-penetrating peptides [65, 66]. In order to better understand the modification of phage capsid proteins with NHS esters, Jin et al. [67] established a generalized kinetic model for protein amine modification to predict the modification level of filamentous phages. It was found that virus particles with 0.03 biotins per pVIII subunit have 50% of the maximal binding capacity for a streptavidin conjugate. Such information can reduce excessive modification of targeted structures in future studies, perhaps allowing additional modifications to be used to append secondary cargo [63].

Except for the widely used NHS esters, amine groups can also be functionalized with Tetrafluorophenyl (TFP) esters and isothiocyanates. TFP ester is a water-soluble reagent that reacts with amines under alkaline pH conditions to form stable amide bonds. Unlike NHS esters, which are easily affected by alkaline hydrolysis, TFP esters show a lower hydrolysis rate under alkaline conditions, thus improving the coupling efficiency [68]. Isocyanates and isothiocyanates react with amino groups to form urea and thiourea. Isothiocyanates are more commonly used for bioconjugation than isocyanates because they are more stable in storage [58]. To achieve site-selective modification, an N-terminal two-step transamination/oxime formation strategy has been developed. The phage was functionalized with pyridoxal 5' phosphate (PLP), in which the N-terminal amino group was converted to a pyruvamide group, which was then converted to an oxime using alkoxyamine reagents. This chemistry reaction has been shown to be highly selective for the N-terminal groups without transamination of lysine ε-aminos [69].

Carboxylate groups are present at the C-terminus of proteins and on the side chains of aspartic acid and glutamic acid, carboxylates are less reactive in water, so they are usually activated by carbodiimide crosslinkers, such as water-soluble 1-ethyl-3-(3-(dimethylamino)propyl)-carbodiimide hydrochloride (EDC), to form a reactive O-acylisourea intermediate. Then form stable amide bonds with primary amines under the mild acid environment. Furthermore, adding NHS ester can form a more stable intermediate, thereby increasing the efficiency of EDC coupling reaction [58, 70]. Based on this chemical reaction principle, the amino group on the cationic photosensitizer Nile blue dyes (NB) can be successfully coupled with the carboxyl group on the phage head capsid protein through EDC/NHS reaction [71]. Chloramphenicol, a poorly water-soluble drug, effectively binds to phage capsid protein through hydrophilic aminoglycoside antibiotics by using EDC coupling strategy [72]. In addition, glutaric anhydride has been conjugated to primary amines on M13 phage to increase the number of carboxylate groups available for polymer immobilization in downstream reactions [73]. EDC coupling, a chemical modification strategy, can be effectively used to target carboxylic acid moieties on phages to generate novel phage materials. Because EDC coupling is not a site-specific chemical modification strategy, any carboxyl entity on the displayed protein is easily modified [63].

Thiol groups are present in the side chains of cysteine. Cysteine is commonly used as an alkylation site for electrophilic halides or Maleimide [70]. Maleimide reacts with thiols under mild pH and temperature conditions to form stable thioether bonds. On this basis, a cysteine residue (C87) is added to each monomer sequence of the MS2 capsid protein to allow simple modification of the capsid interior with maleimide reagents [74]. Introducing thiol groups to noble metal surfaces is a well-known method to induce metal-peptide conjugation. Thiol groups readily form bonds with metal ions and materials. Cysteine residues have been incorporated into the fd phage capsid to improve binding with gold materials [51, 58]. Chemical modification of phages can also be used in targeted therapy. For example, Peng et al. [75] developed a scheme, N-succinimidyl-S acetylthiopropionate (SATP) has been chemically modified with M13 phage capsid to introduce thiol groups, and gold nanorods are bound to the phage via thiol-gold bonds. Target bacteria are specifically captured by phages and modified with gold nanorods for photothermal therapy.

Phenolic groups are present on tyrosine and histidine residues. Diazo groups are known to react with a variety of protein functional groups, including tyrosine, histidine or lysine residues. Thus, diazo compounds can be used for modification to form diazo-linked conjugates. Adjusting the pH to 7 favors the reaction with histidine imidazole groups, and these highly reactive diazo groups can be used to modify tyrosine residues by electrophilic attack on the active tyrosine pi system at pH typically greater than 8.5 [58, 63]. Based on this chemical modification principle, there are 180 tyrosine residues (tyrosine 85) available for modification in the internal cavity of MS2 phage, the phage is exposed to twofold excess nitroazo salt, tyrosine groups of MS2 phage can be extensively modified in a short time [76]. Besides, Murugesan et al. [77] modified M13 phage through genetic engineering, fused tyrosine residues accurately with the main coat protein and specifically react with aromatic amines via diazotization reaction. The prepared azo-M13-phage nanowires exhibit reversible photo-responsive properties.

Aldehydes are universal functional groups for protein functionalization [78], such as glutaraldehyde (GA), which is usually used to modify phages to produce chemically and thermally stable cross-linked biomaterials. Glutaraldehyde can react with several nucleophilic functional groups existing in proteins, including amines, mercaptans, phenols and imidazoles, among which lysine ε-amino reacts best with glutaraldehyde [79]. Phages are chemically modified to confer functionality, providing the desired handle for further modification. It is worth mentioning that a series of bioorthogonal reactions have been introduced by modifying N-terminal amines to aldehydes [59]. An important example is the periodate oxidation of N-terminal serine and threonine residues. N-Terminal serine and threonine residues contain a β-amino alcohol motif that can be specifically oxidatively cleaved by sodium periodate to generate an aldehyde handle. This handle allows for a second reaction to add the desired conjugate. The oxime reaction has been used in aldehyde displaying phages to attach aminooxy conjugates [58]. Kitov et al. [78] described the rapid coupling reaction of 2-aminobenzylamine oxime (ABAO) derivatives with aldehydes to bind materials to M13 phage. It was expected that this reaction would serve as a platform for developing new bioconjugation strategies, fluorescent probes, and post translational diversification of genetically-encoded libraries.

Non-natural amino acids can also be inserted into the phage capsid protein to exert its function. Because many kinds of amino acid residues on phage capsid protein may have the same reaction group, cross-reaction will inevitably occur. Unnatural amino acids can be introduced to achieve site-specific modification [70]. Sandman et al. [80] integrated selenocysteine into peptides displayed by M13 phage using a natural selenocysteine opal suppressing tRNA. Due to the ability of selenocysteine to exhibit stronger nucleophilicity and reactivity than cysteine under physiological pH conditions. Therefore, it allows for selective binding with small molecule reagents [81]. A variety of unusual or unnatural amino acids have been synthesized, which contain azido or alkynyl groups capable of undergoing a copper-catalyzed azide − alkyne cycloaddition click chemistry reaction [58]. Using residue-specific unnatural amino acid incorporation, azide-containing unnatural amino acids displayed on M13 phage have facilitated conjugation to alkyne-functionalized fluorophores and gold particles [82, 83]. In addition, a non-natural amino acid, p-aminophenylalanine (paF), was introduced into the surface of MS2 phage. It can react with phage capsid via oxidative coupling mediated by sodium periodate. This method has been used to modify MS2 phage-like particles and show selectivity, even in the presence of tyrosine [74]. Recently, Wang et al. [84] encoded N-acryloyllysine (AcrK) gene in a phage-displayed peptide library. The displayed peptides were cyclized via a proximity-driven Michael addition reaction between cysteine and amber codon encoded AcrK, which solved the problem encountered in cysteine conjugation. As a supplement to phage display technology, this novel method will be widely used in drug discovery.

In conclusion, the chemical modification method of phage-drug coupling has improved the deficiencies in the process of drug use. Various chemical conjugation strategies have shown great potential in the development of new nanomedicines for targeted drug delivery, while providing the possibility of reintroducing non-specific drugs hitherto excluded from use as therapeutic agents. A variety of promising strategies for chemically modifying phages have been developed, but most of these techniques lack the high degree of specificity and control required for many applications. Although chemical modification is simple, effective and direct, such treatment may also lead to cross-linking between viral particles, denaturing proteins or changing the characteristics of phages. Whether this will happen in practice must be analyzed specifically, but the phage itself seems to be significantly tolerant to chemical modification [85].

#### Genetically engineered phages as delivery vehicles

Phage virus particles are composed of capsid proteins that encapsulate DNA or RNA genome [13]. It has the stability in a certain pH range and the ability to resist the degradation of nuclease. Coat proteins protect the virus genome from degradation after injection, thus providing protection for valuable gene sequences [92]. The phage provides a vector with engineering specificity for gene delivery. It is a promising method to integrate the target gene into its genome and deliver it to eukaryotic cells. The phage has a strong cloning ability to carry a large amount of exogenous DNA. For example, lambda phage has a DNA cloning ability of about 20 kilobase pairs (kbp), which is much higher than the maximum of 5 kbp of plasmid DNA vaccine [93]. Compared with other non-viral gene delivery vectors, the significant advantage of phage lies in its inherent ability to effectively protect RNA [94]. The use of RNA phages such as MS2 has proven to be an effective RNA delivery method. For example, Prel et al. [95] recombined HIV-1-derived lentiviral RNA in MS2 phage, enabling efficient gene delivery to alter the expression of osteogenic transcription factors encoded by mRNA in bone marrow mesenchymal stem cells.

**Fig. 2:**
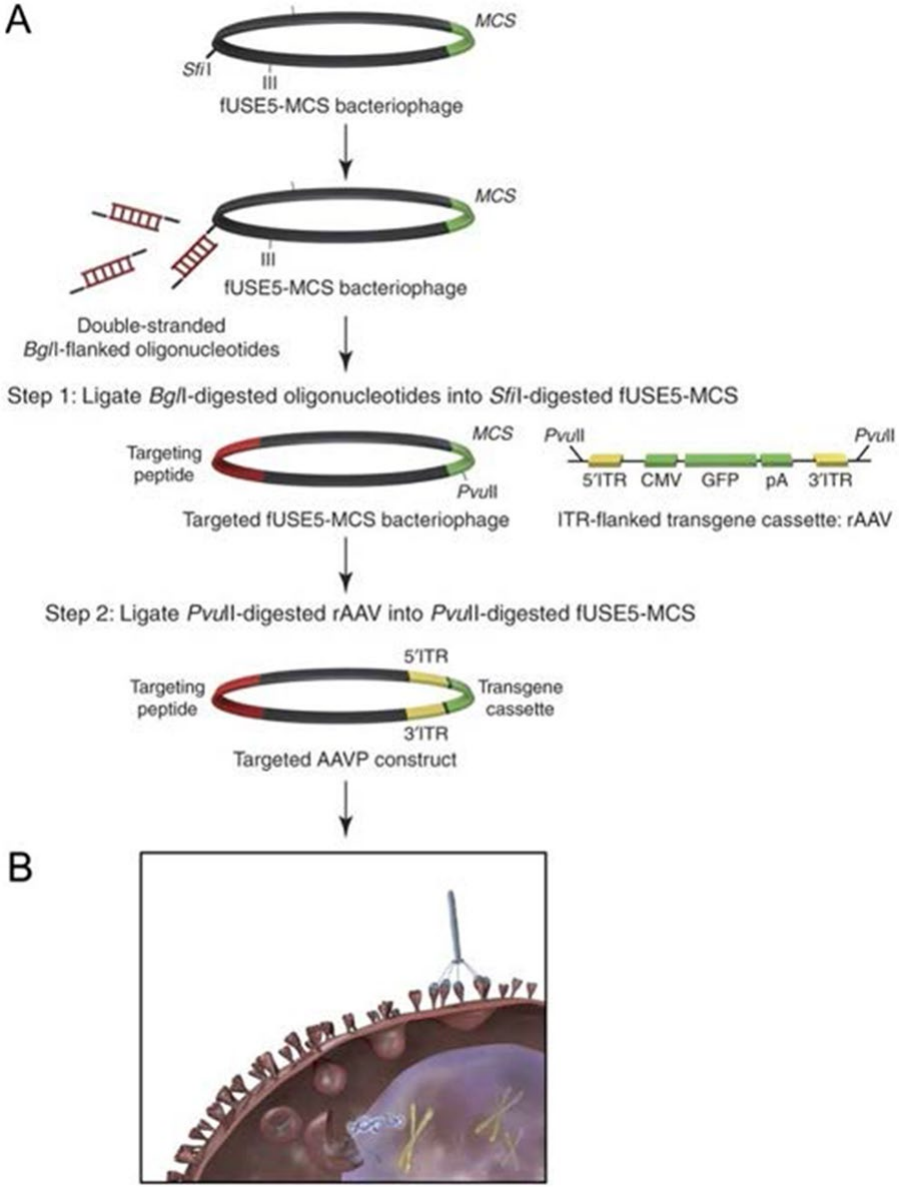
**A** Cloning scheme for generating targeted AAVP and control vectors. **B** Targeted AAVP particles bind to specific cell-surface receptors in target tissues and internalization after systemic administration. Reprinted from Ref. [98]

Although phage-mediated gene delivery holds great promise, most notably low transduction efficiency severely limits its transformational applications [96]. To date, some research groups have reported various gene modification methods to improve the gene transduction efficiency of phages. Hajitou et al. [97, 98] introduced the eukaryotic gene cassettes on both sides of the ITR from AAV into the phage genome to hybridize the M13 vector with AAV (called AAVP). AAVP combines the good biological properties of eukaryotic and prokaryotic viruses, which facilitates the delivery of genes to tumor cells and improves the efficiency of gene transduction (Fig. 2). In order to further improve the gene delivery efficiency of the AAVP system, Azadeh Kia et al. [99] proposed a strategy to improve AAVP at the genomic level, by introducing the eukaryotic tumor-specific promoter Grp78 instead of the CMV promoter to drive gene expression, while AAVP combined with Histone deacetylation inhibitors and DNA methylation inhibitors. More recently, Kao et al. [96] designed "TransPhage" through shortening the phage length with the restructured f1 origin, it carries the domain A, domain B, the gene, the packaging signal, and the loop B–C in the described order. TransPhage successfully transduced human cells with excellent efficiency (up to 95%). Future detailed studies of the molecular aspects of the interaction between phage and eukaryotic cells may reveal new ways to improve phage-mediated transduction [100].

Genetic engineering, including phage display, is a powerful tool for using phages to design targeted gene and drug delivery vectors. Phage display technology is to insert foreign peptide or protein gene into phage specific protein gene by means of genetic engineering, and the coding gene is encapsulated by coat protein and displayed for fusion. This method is based on the direct connection between the phage phenotype and its encapsulated genotype. The displayed peptides or proteins can maintain relative spatial structure and biological activity, recognize and bind to target molecules [101, 102]. There are two main methods for genetic modification of phages, namely surface display via phage vector or through phagemid. For some filamentous phages, although using phage vectors to display exogenous peptides has once-and-for-all advantages, the longer the length of the exogenous DNA fragment, the higher the instability of the recombinant phage vector, and it is difficult to reproduce with high purity on a large scale. At this point, using phagemid vector-based display system can partially address these shortcomings [70]. Phage technology has been used to find new protein ligands, such as enzymes and receptors, as well as antigens for tumor diagnosis and targeted therapy [22]. Phages are genetically modified to express foreign proteins specific to specific sites, for example, heterologous targeting peptides derived from biological interactions or evolved from random peptide libraries through in vitro selection of desired receptors can be inserted as fusion insertions with capsid proteins to deliver drugs to specific sites (such as tumor cells) [85]. In addition, the combination of different display methods can provide double display phage particles. This can be achieved by combining modified genes, or through modified phage propagation protocols using double virus infection [59]. Phage display technology promotes the development of vaccines, which provides an opportunity to screen and identify functional peptides or proteins with required immunogenic characteristics [102]. At the same time, the high immunogenicity of phages can be used to enhance the immune response of vaccine delivery.

In summary, phages have unique genetic flexibility. Based on the size and type of target genes, suitable phages can be selected and exogenous genes can be inserted into the phage genome through genetic engineering methods for gene delivery. Phages can also be genetically engineered to accommodate various surface modifications and subsequently display ligands that can target specific cell types, ultimately improving therapeutic efficacy.

### Phage-derived components assisted delivery system

For a long time, people have been seeking suitable targeted ligands to enhance the therapeutic index of drugs by enhancing the penetration and retention effects. Phage display technology that improves efficiency and reduces research costs can display antibodies, peptides, or proteins on the surface of different phages. In particular, peptides are considered an attractive class of targeting ligands. Because of their small size, peptides are better able to penetrate tissues to reach target cells, and many of the problems associated with larger-sized antibodies appear to be resolved with peptides. At the same time, they have the same functions as proteins due to their similar composition [103, 104]. In addition, phage display peptides have the advantages of high immunogenicity, high biocompatibility, high particle carrier loading rate, and can be selected as multifunctional ligands in the biomedical field, such as cell-targeting, tumor-homing, cell-penetrating [105].

Specific peptides screened from phage display libraries and used for targeting can improve the therapeutic index of drugs by reducing side effects and allowing higher drug accumulation at disease sites through direct chemical conjugation with drugs [21]. Du et al. [106] used in vivo phage display technology to identify peptides that can specifically bind to hepatocellular carcinoma (HCC) cells. After coupling with DOX for in vivo targeted therapy, peptide A54 inhibited tumor growth, improved overall survival rate of mice, and did not cause serious side effects. An interesting evolution is that several research groups have integrated phage display technology with nanocarrier-based delivery platform for targeted delivery of drugs and genes. Phage coat protein is an integral membrane protein that tends to spontaneously insert into the lipid bilayer when dissociated from the phage assembly [107]. On this basis, Wang et al. [108] proposed to directly incorporate phage pVIII coat protein fused with tumor specific peptides into the liposomal bilayer of DOX-loaded PEGylated liposomes, phage fusion coat protein would span and anchor the lipid bilayer via its C-terminal hydrophobic helix, allowing N-terminal-specific peptides to be displayed on the surface of the carrier particle. The improved complex resulted in better uptake of DOX into MCF-7 cells and more efficient killing of target cells. Afterwards, they envisioned that the amphiphilic nature of phage fusion coat protein should also enable its stable incorporation into polymeric micelles. Therefore, they tried to construct mixed micelles made of polyethylene glycol-phosphatidylethanolamine (PEG-PE) conjugates and MCF-7-specific phage fusion coat protein to load the hydrophobic drug paclitaxel, further improved tumor targeting efficiency of micellar-encapsulated drugs [107]. The above studies used the amphiphilicity of phage coat protein to avoid chemical modification with nanocarriers, avoiding chemical modification makes the preparation process more complicated and may even change the properties and specificity of peptides [109].

However, specific peptides or proteins identified by phage display technology are typically conjugated with delivery vectors through chemical modification to improve the specific targeting of loaded therapeutic cargo. Nam et al. [110] selected primary cardiomyocyte (PCM) specific peptide by phage display, which was modified by conjugating cysteine-terminated PCM to an activated polymer using a crosslinker. PCM-modified polymers can efficiently and specifically bind cardiomyocytes for siRNA delivery. In addition, phage proteins themselves can be assembled into nanoparticles for gene delivery. Bedi et al. [111] efficiently encapsulated siRNA by phage fusion proteins which display cancer-targeting peptides. The N-terminus of the phage fusion protein is the targeting ligand, while the positively charged C-terminus interacts with the negatively charged phosphate of the siRNA to form phage nanoparticles that deliver siRNA to the target region. The above research indicated that directly inserting peptides into nanocarriers is not a universal method at present, and sometimes chemical modification is inevitable. Peptides or proteins derived from phages represent the driving force to recognize target molecules and transform goods into self-navigation formulations.

Overall, phage display-derived peptides or proteins have key functions as targeted ligands in overcoming obstacles encountered in drug and gene delivery processes, including cell targeting and tissue permeation. They can serve as promising targeted ligands for the preparation of drug formulations, used to functionalize nanocarriers and enhance therapeutic effects.

## Classification of phage-based delivery systems

**Fig. 3:**
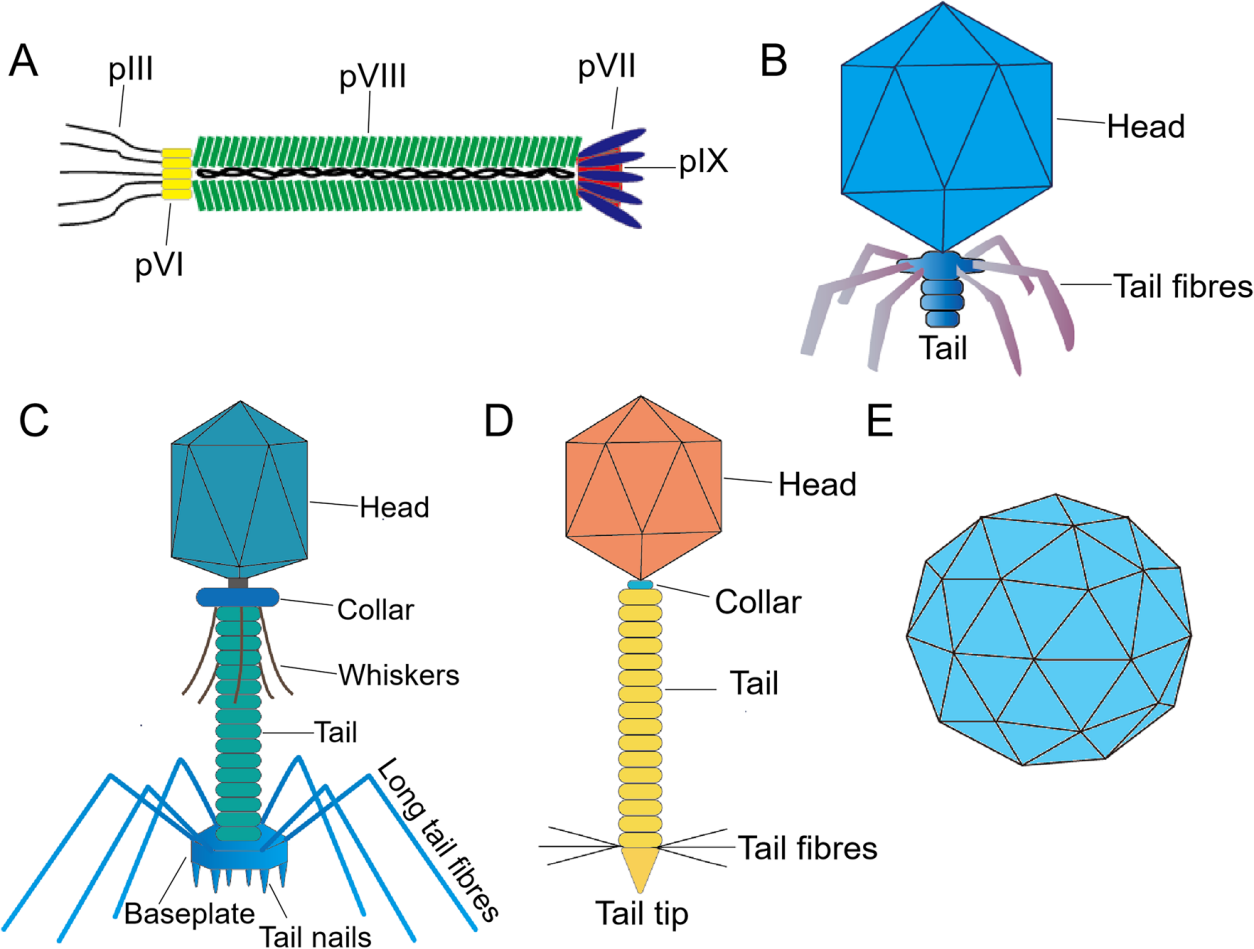
The main types of phages used as nanocarriers in drug and gene delivery. **A** M13 phage is approximately 880 nm in length and 6-7 nm in diameter. It is mainly composed of five capsid proteins (pIII, pVI, pVII, pVIII, pIX) and encapsulated cssDNA. **B** T7 phage has an icosahedral head of 55 nm in diameter, which encapsulates a 40 kbp linear dsDNA, and the tail is 19 nm long, short and uncontracted. **C** T4 phage has an icosahedral head with a length of 120 nm and a width of 86 nm, which encapsulates a 171kbp linear dsDNA, and six tail fibers with a length of 160 nm are attached to the tail. The tail is long and retractable. **D** Lambda phage has an icosahedral head with a radius of approximately 30 nm, which encapsulates a linear 48.5 kbp dsDNA, and a tail with a length of 150 nm. **E** MS2 phage consists of a ssRNA and 180 protein subunits surrounding its genome to form an icosahedral capsid about 27 nm in diameter

Each type of phage varies greatly in many ways, including size, shape and surface proteins most commonly used for antigen display. Targeted ligands display on the surface of phage offer versatility. A large number of drugs and genes can be incorporated into phages through chemical interactions or genetic manipulation. In this section, we describe the major phage species that have been used as carriers for drug and gene delivery (Fig. 3).

### Delivery system based on M13 phage

M13 phage is a filamentous phage with a length of about 880 nm and a diameter of 6–7 nm. It is mainly composed of five coat proteins (pIII, pVI, pVII, pVIII, pIX) and their wrapped 6407 nucleotides length circular single-stranded DNA (cssDNA) [112].The size of the M13 phage is determined by the length of the DNA core, and longer or ultrashort phages can be made by inserting and deleting nucleic acid [113]. In addition, M13 phage is also a temperate phage, which reproduces and expresses in the host bacteria, but does not lyse the host bacteria. As the bacterium replicates, it continually synthesizes progeny phages. Phage particles are secreted from infected cells, and the host cells continue to grow and divide [114]. The characteristic of M13 phage makes it an economical and available source of biomedical material.

Phages are highly immunogenic and can be rapidly recognized by antigen-presenting cells (APCs), inducing immune response against their original coat protein, and the immune response can also be induced by phage DNA. In addition, the deoxycytidine-phosphate-deoxyguanylate (CpG)-rich region of the M13 phage genome can activate the toll-like receptor 9 (TLR9) signaling pathway, thereby enhancing the immune response [115]. Based on the high immunogenicity and good safety of M13 phage, phage display technology can be used to display certain antigenic peptides on the surface of M13 phage to prepare vaccines. Moreover, the surface of M13 phage is negatively charged, which makes it easier for them to adsorb positively charged substances. By simply mixing M13 phage and PEI, negatively charged antigens such as antigenic peptides, proteins and cell membranes can be successfully adsorbed through electrostatic interactions to prepare phage-based vaccine platforms [40].

**Fig. 4:**
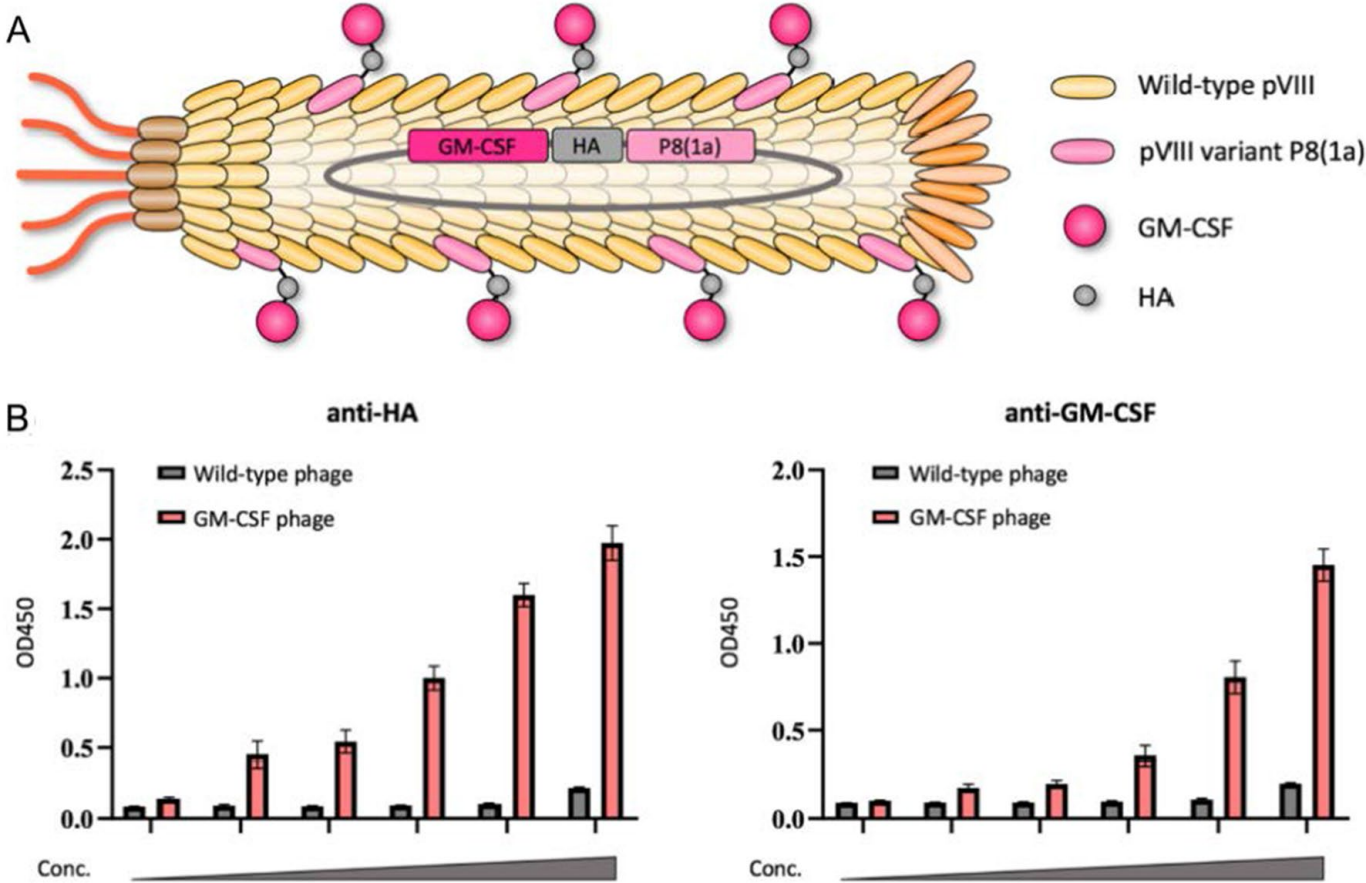
**A** The schematic diagram of the 8 + 8 type phage display for GM-CSF presentation on M13 phage. **B** The display of GM-CSF on the phage was detected by ELISA assays. Use a HA-tag antibody (left) or a GMCSF antibody (right) as the capturing reagent. Reprinted from Ref. [118]

There are many types of phages used in phage display systems, and the most commonly used phage display system is M13 [116]. One of the advantages of filamentous phage display system is that all five coat proteins can display foreign molecules, and it is considered to be one of the most efficient display systems [117]. Due to wide compatibility, major capsid protein pVIII and minor capsid protein pIII are the most commonly developed and modified capsid proteins for peptide display. The minor capsid protein of M13 phage is commonly used to display targeting ligands, and pIII is the most commonly used and stable protein for displaying such ligands [118]. Although the pIII protein has only 3–5 copies, it allows the insertion of protein sequences containing more than 100 amino acids [119]. Likewise, two other minor capsid proteins, pVII and pIX, located opposite pIII, have also been used to display targeting molecules such as peptides or antibodies [120, 121]. The pVIII protein is the major capsid protein of M13 phage, located on the sidewall of filamentous phage. pVIII protein is most commonly used due to its high copy number, but it can only tolerate a small peptide (about 10 amino acids) [118] as it must pass through the cell membrane, and only when the helper phage provides the pVIII protein, a larger peptide chain can be displayed at the N-terminus, otherwise it will affect the phage assembly [22]. Wang et al. [118] employed the "8 + 8" type phage display system to display the large anti-tumor protein GM-CSF with multiple copies on the major capsid protein through the highly tolerant pVIII variant P8(1a), solving the low tolerance problem of wild-type pVIII displaying only small peptides (Fig. 4).

In order to enhance the effectiveness of drugs targeting specific cells and avoid unnecessary side effects on non-target cells [122], M13 phages act as nanocarriers to enhance drug delivery in two ways: one way is to display targeting peptides or antibodies to enhance targeted delivery. M13 phage is a chemically coupled high-density carrier, and the main capsid protein pVIII is an ideal target for chemical modification of drugs due to its dominant quantity. Chemical groups such as amino, carboxyl, and phenolic groups on phage can be used as linkers for modifying drugs [60]. Therefore, by displaying targeting ligands on the phage minor capsid protein and simultaneously loading therapeutic agents on the phage major capsid protein, physiological barriers can be overcome to improve its therapeutic effect. For example, Wang et al. [118] and Ghosh et al. [20] have both displayed cancer cell-targeting ligands on the minor capsid protein while loading therapeutic agents on different phage display sites to target colorectal and prostate cancer, respectively. Another way to enhance drug delivery is to use the nanofiber-like thin structure to penetrate barriers, especially in the brain. The engineerability, rod-like structure and high aspect ratio of M13 phage can enhance functionalization, cell uptake and transport. Compared with spherical materials, they tend to marginalize and migrate along the vascular wall [123, 124]. These characteristics make it an ideal system for transporting goods across the blood–brain barrier, which makes it difficult for exogenous therapeutics to enter the brain, and can be used as a target for nanotherapy in the central nervous system [116, 125]. The linear shape and structure of M13 phage allow particles to penetrate the blood–brain barrier (BBB). In order to further deliver drugs to the nervous system, Tsedev et al. [126] cracked the M13 assembly system. The innovative inho system produced ultrashort phages by packaging the designer cssDNA genomes with different sequence lengths. The inho phages with 50 nm length overcome the challenge that many nanoparticles must reach the required intracellular and tissue penetration depth to further enhance the targeted delivery of drugs.

M13 phage has also been developed as a gene delivery vehicle. By displaying targeting ligands on pIII and introducing target genes into phages, engineered phages can successfully deliver genes to mammalian cells [127]. It is worth mentioning that AAVP is a promising tool for safe and efficient gene transfer. Initially, Hajitou et al. [97, 98] proposed to display the selected ligand peptide RGD-4C on the coat protein pIII of phage, then, the eukaryotic gene cassettes on both sides of the ITR from AAV were introduced into the phage genome to form RGD-4C AAVP. This ligand-directed AAVP facilitates gene delivery to tumor cells and improves gene transduction efficiency. Since then, AAVP vector has been used to enhance the delivery and expression of various genes [128–130]. More recently, Kao et al. [96] discovered important factors that significantly impact on phage transduction. That is, the up-regulation of PrimPol or down-regulation of DMBT1 can significantly improve the efficiency of phage transduction. In addition, they designed TransPhage with minimal length and optimal transduction efficiency to successfully transduce human cells with excellent efficiency (up to 95%). So far, using phage as a delivery system to introduce genes into animal cells is a promising design, and the key issue is to find the best solution to improve transduction efficiency [22]. Finding alternative methods for endocytosis or proteasomal-independent intracellular trafficking may further improve the transduction efficiency of vectors [131].

Recently, hydrogels have attracted wide attention in biomedical applications due to their biocompatibility and structural versatility [132]. As promising biological building blocks, M13 phages can be used to construct bioactive materials for biomedical applications [133].Tian et al. [134] developed a bioactive gel system based on M13 phage by chemical cross-linking. M13 phage was crosslinked with GA and EDC, respectively, resulting in the gelation of phage aqueous suspension. They embed virulent phages into the gel to enhance the bactericidal ability of the gel, which was expected to be used to combat multi-drug resistant (MDR) bacterial infections. Based on the significant advantages of M13 phage gel, Dong et al. [54] proposed to apply it to antitumor therapy to reverse immunosuppressive tumor microenvironment (TME). M13 phage gel was constructed by chemical cross-linking GA and phage capsid protein by Schiff base reaction. PdNPs were synthesized on pVIII capsid protein in situ and NLG919 was further loaded into a gel. This M13 phage-based bioactive gel system not only had a strong loading capacity, but also acted as an antigen library and natural adjuvant to induce the activation of immune cells. Besides, the loaded drugs can be continuously released at the target site, achieving long-lasting therapeutic effects.

Although the application of M13 phage as a delivery system is interesting, there are still the following limitations that need to be further addressed. Firstly, the displayed peptides are restricted. The M13 phage is expressed and assembled within the bacterial host, secreted without damaging the host cell, thus limiting the type, length, and quantity of displayed peptides [116]. Secondly, the safety concerns. Although M13 phages do not cause cellular lysis, they are immunogenic viruses that can stimulate the immune response in the body. In patients at high risk, therefore, the use of phages may be limited. Furthermore, since M13 phages are constructed in bacterial hosts, they may contain different levels of LPS, it is necessary to develop more effective purification systems for future research to reduce these LPS [135]. Nevertheless, the use of M13 phages as delivery system has demonstrated their potential for application in various clinical environments.

### Delivery system based on T7 phage

T7 phage has structural symmetry with an icosahedral head of 55 nm in diameter and a 19 nm long tail, which encapsulates a 40 kbp linear double-stranded DNA (dsDNA) and a short, non-contracting tail. It consists of six major proteins:gp10A, gp10B, gp8, gp11, gp12, and gp17. The two major coat proteins include gp10A and gp10B, with six fibers attached to the end of the tail, each consisting of the gp17 protein [135–137]. T7 is a lytic phage, which is released directly from the host cell after assembling and lysing it in the cytoplasm. Therefore, the proteins displayed on the surface of T7 phage do not need to be secreted through the host bacterial membrane, compared to M13 phage, it has the ability to display a large polypeptide or even a protein [138]. T7 phage are very stable and do not affect their genome stability even when inserting more than 1 kb of exogenous genes [137]. Xu et al. [139] found that T7 phages can tolerate 2 kb of exogenous gene insertion and still maintain their structural integrity, and the study demonstrated that T7 phages can be an effective carrier for DNA vaccine delivery. T7 phage grow rapidly and form plaques within 3 h, saving a lot of cloning and screening time. The high replication rate of T7 also means that it can be used for phage therapy to effectively control bacterial infections. The titer of phage increases only in the presence of host bacteria, thus providing a self-limiting system to control infection [135]. Moreover, T7 phages remain very stable under extreme conditions where other phages cannot survive (such as high acid concentration or temperature), which facilitates efficient high-throughput biological screening [136].

In the T7 phage display system, foreign peptides can be fused into phage head capsid proteins gp10A and gp10B. Because the 10B region exists on the surface of the phage, the foreign gene is usually inserted into the C-terminal P10B by phage display technology [136]. Besides, foreign gene sequences can be inserted into other regions, such as gp17 [140]. The peptide-encoding gene fragment is typically inserted into the gp17 gene, while the protein-encoding gene fragment is typically inserted into the gp10B gene [22]. The ratio of the two proteins, the major coat proteins gp10A and gp10B, can be altered to construct a functional coat. Thus, the T7 phage display system can adapt to changes in peptide or protein sequences, thereby displaying high copy number small peptides or large proteins with low to medium copy numbers. Interestingly, the antigen density displayed on granular materials has been shown to affect the type of immune response produced, indicating a subtle balance between using phages to display antigens at low or high copy numbers [46, 117].

The size and shape of T7 phage are ideal for intracellular delivery of therapeutic agents. Wong et al. [141] proposed that the addition of a targeting ligand consisting of 33 amino acids to P17 could target proteins, polymers, siRNA and particles to hepatocytes. Another important advantage of T7 phage as a targeting vector is that relatively long peptides can be displayed on the major coat protein. For example, Oh et al. [52] constructed a recombinant T7 phage whose capsid protein displayed both gold-binding and prostate cancer cell-binding peptides. Then, gold nanoparticles (AuNPs) with photothermal effect are combined to form AuNP clusters. The study demonstrated that targeted AuNP clusters rapidly kill prostate cancer cells under low-intensity light irradiation. Furthermore, T7 phage itself as a lytic phage coupled with the maturity of genetic engineering technology makes it a potential candidate for targeted antibacterial therapy, and T7 phage has been considered as a potential method to solve the bacterial resistance caused by traditional antibacterial drugs [142]. It has been reported that the engineered T7 phage expressed AiiA lactonase can effectively degrade acyl to serine lactones (AHLs) from many bacteria. At the same time, it effectively inhibited biofilm formation [143]. Although T7 phages caused much less bacterial lysis than antibiotics, the lysis was sufficient for targeted antimicrobial therapy. The problem that needs to be solved in the future is whether it is possible to establish an ideal drug delivery pathway using multiple phage mixtures and modify their genes to inactivate bacterial resistance genes [136].

In short, T7 phage is suitable for engineering, tolerating long exogenous gene sequences into its genome, and easy to display affinity peptides [144], which has the potential to be used directly in combination with photodynamic, photothermal, or gene therapy [136].

### Delivery system based on T4 phage

T4 phage is a relatively large virus with an elongated icosahedral head 120 nm long and 86 nm wide, and a long and retractable tail. The head encapsulates approximately 171 kbp of linear dsDNA, with six 160 nm long tail fibers attached to the tail. T4 capsid proteins are divided into two main groups, including the major essential capsid proteins, gp23, gp24, gp20, and there are also non-essential coat proteins, the highly outer capsid protein (HOC) and the small outer capsid protein (SOC), with high antigenicity [145–147]. Therefore, foreign antigens are usually displayed on Hoc or Soc for vaccine preparation.

Phage-based vaccines have the unique advantages of simple and rapid modification [117]. T4 phage is a promising vector for vaccine delivery. Tao et al. [148] prepared a plague vaccine using T4 phage as a nanoparticle delivery system. By fusing the mutated F1 with the V antigen to obtain the F1mut-V protein. The F1mut-V protein antigen was then fused to the Soc on the T4 phage. Recombinant T4 phage provided full protection against Yersinia pestis attack in two rodent models. In addition, the main advantage of T4 phage over other phages is the possibility of simultaneous display of SOC sites and HOC sites and the ability to display a larger number of copies. Dual site display of T4 phage can induce more effective immune response at high levels of immunogenic exposure [149]. Furthermore, targeting molecules can be fused to Hoc or Soc for specific targeting cells, and in vivo targeted therapies can be customized by simply changing the targeting ligand. This is a significant advantage for T4 and phage-based delivery systems compared to other delivery vectors [150]. Compared with the large number of research reports on T4 phage designed as vaccines, there are fewer reports on the delivery of functional proteins to tumor sites by T4 phage for tumor treatment. More recently, Hou et al. [151] used T4 phage as a delivery vehicle, and the catalase (Cat) protein was displayed on the surface of the phage through the interaction of Soc and T4 surface capsid protein. More importantly, the number of displayed Cat can be precisely controlled by feed concentration. Additionally, the photosensitizer is attached to the surface of the T4 phage via chemical conjugation. The prepared "super tumor phage" can alleviate tumor hypoxia, providing a new strategy for enhancing PDT to alleviate tumor hypoxia.

**Fig. 5:**
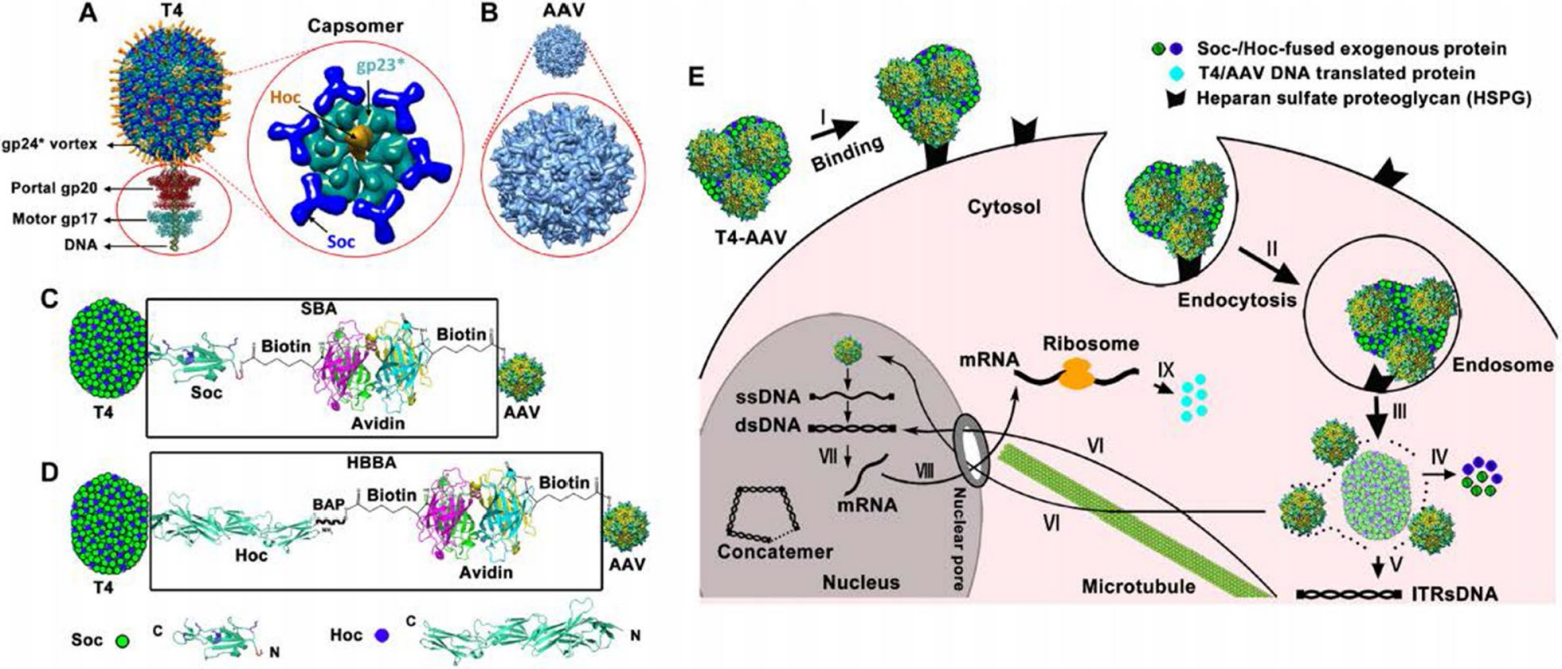
The construction strategy of the T4-AAV hybrid vector and the schematic diagram of its delivery to mammalian cells. **A** T4 DNA packaging and protein display machine. **B** AAV DJ subunits assemble to form the icosahedrally symmetric virus capsid. **C** and **D** Design principles of the T4-AAV vector. Conjugating the T4 head with biotin-labeled AAV by avidinbiotin interaction. **E** Schematic diagram of protein and gene delivery into mammalian cells by the T4-AAV vector. Reprinted from Ref. [153]

The DNA packaging mechanism of the T4 phage is very rapid and robust (packaging rate up to 2000 bp/s), providing the largest exogenous DNA (up to 170 kb) and protein (up to 1025 molecules) payload [152]. The T4 phage packaging machine consists of two main components, the head and the packaging motor (gp17). The T4 motor lacks sequence specificity and starts the packaging at any end of the DNA. Therefore, any exogenous DNA can be packaged in the T4 capsid until the head is full and there is no length requirement [150]. Based on these findings, Tao et al. [152] proposed to reconfigure the phage packaging machine to deliver genes and proteins to mammalian cells. In their research, combinations of reporter genes, vaccine genes, functional enzymes and targeted ligands can be integrated into the T4 head and delivered to intracellular or targeted antigen-presenting dendritic cells with nearly 100% efficiency. T4 and many phages have inherent size advantages and are suitable for deep engineering to create and design recombinant phages. However, phages do not have evolved mechanisms for entry into human cells and intracellular trafficking [150]. Therefore, integrating the complementary characteristics of T4 and AAV is an ideal choice for many therapeutic applications. Zhu et al. [153] designed a hybrid virus vector composed of phage T4 and AAV. The AAV was attached to T4 head through avidin–biotin cross-bridges using the phage decoration proteins Soc and Hoc. By virtue of its natural ability to enter human cells, AVV acts as an efficient driver to deliver each relevant cargo into mammalian cells (Fig. 5). After that, they further modified the T4-AVV platform. Taking advantage of the high anionic characteristics of the surface of T4 capsid, cationic lipids can spontaneously bind to T4 capsid. The modified T4-AVV, with its positively charged lipid coat, was able to bind to the negatively charged and lipophilic surface of human cells and efficiently enter human cells, ultimately increasing the transduction efficiency [154].

Overall, the T4 system is attractive not only because of its huge genetic capacity, but also because it does not infect mammalian cells, is non-toxic, and has no pre-existing immunity in the host [150]. In the future, given the inspiration of the T4-AVV platform, new combinations of prokaryotic and eukaryotic viruses, bacteria or synthetic nanoparticles for different applications can be further created. These hybrid vectors with collaborative and superior delivery properties may be used to create future gene and protein therapies for diseases [153]. More importantly, safety problems may arise when T4-AVV is transferred to the clinic, such as adverse reactions or off-target effects that may occur in the host immune system [154]. In the future, further research is needed in order to realize the transformation of phage delivery technology from laboratory to clinic as soon as possible.

### Delivery system based on lambda phage

Lambda phage consists of three main parts; head, tail and tail nanofiber. The head is an icosahedral capsid with a radius of about 30 nm, and the capsid is composed of two main proteins gpE and gpD, which encapsulates a linear 48.5kbp dsDNA. The dsDNA has a sticky end containing 12 nucleotides at both ends of the molecule, so it can be glued into a circular molecule. The length of the tail is 150 nm. Its tail consists of 32 discs, each of which consists of six copies of the tail protein pV [117, 135, 155]. Lambda phage belongs to temperate phage, but it has the common qualities of lytic phage and temperate phage. It can follow a lytic or lysogenic life cycle [92]. As an effective delivery vehicle, this part discusses the latest progress of some key applications of lambda phage as a delivery system, including vaccine delivery and gene transfer.

In the lambda phage display system, gpD and pV proteins are commonly used to fuse foreign peptides or proteins. Early studies of lambda phage fusion peptides were performed by fusing peptides or proteins to the C-terminus of the tail protein gpV [156]. The C-terminus of pV is not essential, and being replaced by foreign peptides or proteins will not significantly affect phage proliferation. Nowadays, the N- or C-terminus of gpD is more common as a display site, with up to 420 copies on the phage and display on it will not affect the function of the phage or prevent the fusion protein from properly binding to the capsid [157]. Pavoni et al. [158] [72] introduced a strategy to display two large proteins simultaneously in the head and tail of lambda phage, using anti-CEA scFv fragment as targeted part to modify gpD and green fluorescent protein or alkaline phosphatase to modify gpV. The results demonstrated the feasibility and potential utility of the phage lambda double display system for use in biomedical applications, which may open the way for the production of novel targeted nanoparticles for diagnosis and treatment.

The lambda phage capsid encapsulates a 48.5 kbp genome, and the huge genome enables the ability to insert foreign genes encoding various vaccine antigens. Lambda phage allows many different vaccines or multiple inserts of the same type to be included in a single phage particle, indicating the great potential of lambda phage as a means of vaccine delivery [58]. Large capacity also means that phage vaccines can contain adjuvant systems such as cytokines genes. It also allows the insertion of genes containing large introns, so phage vectors are very suitable for manufacturing vaccines against eukaryotic parasites. Additionally, the lambda phage delivery system is very robust. According to reports, even at 4 and – 70 °C, no decrease in titre was observed within 6 months, and phage stability was not affected by freezing or thawing [93]. In phages, lambda phage is considered a good candidate for the delivery of DNA vaccines to eukaryotic cells [159]. For example, Clark et al. [160] used lambda phage to construct phage DNA vaccine by expressing hepatitis B small surface antigen (HBsAg). Phage-mediated DNA vaccination produced higher levels of antibodies in rabbits than did commercial vaccines. Studies have shown that the display level of fusion peptides on the surface of lambda phage is much higher than that of M13 phage. Besides, some peptides that are difficult to secrete through the membrane in the filamentous phage display system can also be displayed on the surface of lambda phage [46], expanding the application range of the phage display system.

Gene therapy with lambda phage began in 1971 when Merril et al. [161] delivered the galactosyltransferase gene into human fibroblasts isolated from patients with genetic defects. Thereafter, Lankes et al. [162] displayed an integrin-binding peptide on lambda phage, and display of the integrin-binding peptide increased cellular internalization of phage in vitro and enhanced phage-mediated gene delivery in vivo. Thus, surface modifications that enhance phage uptake could facilitate more efficient gene delivery in vivo. Sapinoro et al. [37] proposed an antibody-dependent enhancement method to increase the transformation of phage-encapsulated genes into target cells, thereby improving the efficiency of phage-mediated gene transfer to positive mammalian cells. However, this approach requires pre-immunization and also requires the presence of specific receptors on the target cells.

In general, lambda phage as a gene and vaccine delivery carrier has a variety of attractive characteristics, high stability, high production capacity, compatibility of rapid and cheap production or purification methods, and inherent biosafety [163]. The ability of lambda phage to carry large nucleic acid sequences and the ability of capsid proteins to be modified with a variety of ligands expand the application range of phages. However, lambda phages have common qualities of both lysis phages and temperate phages, and may undergo lysogeny and lysis. This complex biological characteristic makes the titer of lambda phage lower than that of filamentous phage. In addition, lambda phage has a larger genome, which makes gene manipulation more complex [46].

### Delivery system based on MS2 phage

MS2 phage is an RNA virus with a symmetrical icosahedral capsid structure formed by a single-stranded RNA (ssRNA) and 180 protein subunits surrounding its genome. The diameter is about 27 nm, arranged into an icosahedron with a triangulation fraction T = 3, which protects the inner genomic RNA [164]. The MS2 genome is one of the smallest known genomes, consisting of 3569 nucleotides. It encodes only four proteins: mature protein (protein A), cleavage protein, coat protein (CP) and replicase protein [165].

MS2 phages are easy to produce, their capsids can be genetically and chemically modified, and after removal of their genome, they are able to encapsulate other modified cargoes such as RNA [166]. These properties of MS2 phages suggest that they are good candidates as drug delivery systems. Due to the relative ease of purifying MS2 phage capsids through the E.coli expression system, researchers often use their functionalized capsids as virus-like particles (VLPs) [135]. VLPs are nanoparticles that lack viral genetic material and have a similar external structure and antigenicity to native viruses. MS2 VLP represents a novel delivery platform [167]. MS2 VLPs are small in size and special in shape, capable of packaging and delivering nucleic acids, epitope peptides, and drugs inside phage capsids. They also have excellent adjuvant properties, thereby inducing immune response. It is safer and more effective for vaccine preparation than traditional attenuated or inactivated vaccines. Furthermore, they enable tissue-specific targeting after modification with ligands, a property that can ensure more efficient delivery of targeted drugs [168].

RNA must overcome several obstacles in vivo to function effectively, including rapid enzyme degradation, poor cellular uptake, and poor delivery efficiency to tumors, limiting its practical application [169]. MS2 VLP may be a promising nanocarrier for RNA delivery. The MS2 capsid specifically interacts with the 19-nucleotide RNA stem loop (pac site), which can wrap the target RNA located at the 5’ end of the pac site [170], and can protect the target RNA from being degraded by nucleases. On this basis, Li et al. [171] constructed a mRNA vaccine against prostate cancer (PCa). PAP is the target antigen of PCa vaccine and GM-CSF protein has excellent adjuvant properties. MS2 VLP-based mRNA vaccines against PCa induced strong humoral and cellular immune responses, delaying tumor growth.

MS2 VLPs can be designed to deliver epitope peptides for clinical purposes. When MS2 VLPs are used as the delivery platform for epitope peptides, in most cases, it is realized by modifying the VLP gene sequence through genetic engineering, so that the fusion proteins of VLP components and foreign epitopes are assembled into VLPs during the expression process [168]. One known clinical application of VLP vaccine is to prevent foot-and-mouth disease virus (FMDV). Dong et al. [172] inserted the gene encoding the 141–160 epitope (EP_141-160_) peptide of VP1 into the CP gene of MS2 to prepare CP-EP_141-160_ VLP vaccine, which produced high titers of neutralizing antibodies that protect most animals from FMDV.

**Fig. 6:**
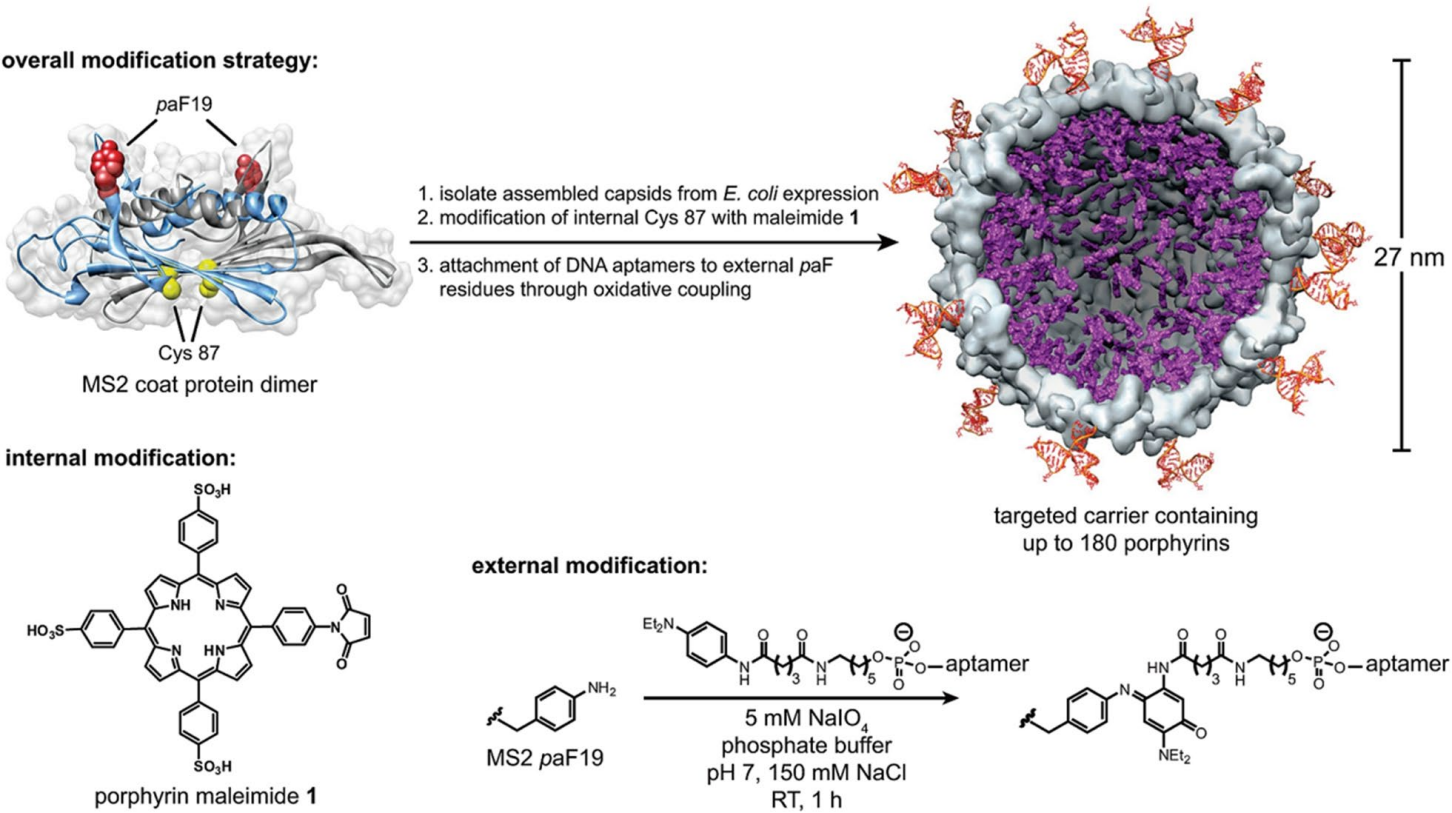
Construction of a multivalent cell-targeted photodynamic therapy carrier by double modification of MS2 coat protein. Porphyrin maleimide 1 (rendered in purple) was used to modify cysteine residues on the capsid interior to attach porphyrin to the inner surface, and the phenylene diamine modified DNA aptamer was oxidatively coupled to the exterior paF using the Schultz amber suppression technique. Reprinted from Ref. [74]

Morphologically, MS2 VLP is a hollow sphere with a diameter of 27 nm and 32 pores. These 2 nm-wide pores allow the installation of small molecules on the inner surface without disassembly [173]. Based on this property, MS2 VLP has important advantages as a drug delivery platform. Drug molecules can be chemically modified both on the outer surface of the capsid and attached to the inner surface of the capsid to prevent cargo degradation and non-specific interactions with normal tissues. In addition, MS2 VLPs have a relatively large internal volume, and even for drugs with above-average molecular weight, MS2 VLPs have sufficient lumen volume [174]. Ashley et al. [175] used liver cancer-specific targeting peptide (SP94)-modified MS2 VLPs to selectively deliver nanoparticles, chemotherapeutic drugs, siRNA cocktails, and protein toxins to human liver cancer cells. Stephanopoulos et al. [74] constructed a therapeutic nanocarrier for targeting Jurkat leukemia T cells. They conjugated cell-specific DNA aptamers to the outer surface of MS2 phage and attached porphyrins to the inner surface. DNA aptamers modify the outer capsid to achieve efficient and selective cell targeting. Porphyrins generate a large number of reactive oxygen free radicals through the photodynamic effect under light and killing a large number of Jurkat cells (Fig. 6). This study demonstrated the great potential of using the double-modified MS2 capsid as a vehicle for targeted therapy.

In short, MS2 VLP is a powerful and versatile delivery platform. MS2 capsid can undergo simple, site-selective double surface modification. Despite a high level of modification, the capsid remains assembled, making it a unique drug delivery carrier [176]. It can not only deliver various drugs with good safety and strong immunogenicity, but also ensure tissue-specific targeting. Therefore, MS2 VLPs have a wide range of practical application prospects.

In summary, phages are widely used as delivery vehicles in the fields of biotechnology and nanotechnology. Phages such as M13, T7, T4, lambda, and MS2 are widely used for different research needs, each with its own advantages and limitations. The M13 phage genome is relatively short and can only carry short foreign gene fragments. Its major capsid protein pVIII is the most commonly used due to its high copy number, but it can only display short peptides. In contrast, the minor capsid protein pIII can display large proteins, but at a much lower density. More importantly, the designed ultra-short M13 phage can become a good nanomaterial. Due to its small size, the relative effective collision efficiency between the target site and the target is greatly improved, so the efficiency of targeting the target is greatly improved. Compared to other phages, its unique linear shape and structure allows it to penetrate barriers, especially in the brain, and could be used as a nanotherapeutic for the central nervous system. In contrast, T7 phage can carry medium-length foreign gene fragments. T7 phage is extremely powerful and its proliferation rate is faster than other phages. In addition, its efficient transcriptional mechanism can achieve a high level of foreign gene expression, while the expression level of other phages is relatively low. Functional coating can be constructed by changing the ratio of the major coat proteins gp10A and gp10B. Therefore, the T7 phage display system can adapt to changes in peptide or protein sequences, thereby displaying high copy number small peptides or large proteins with low to medium copy numbers. T4 and lambda phages have larger gene capacities and can carry longer foreign gene fragments. The main advantage of T4 phage over other phages is the ability to simultaneously display SOC sites and HOC sites, which are unique structures of T4 phage, so it has the ability to display a higher number of copies. More importantly, its DNA packaging mechanism is very fast and robust (packaging rate up to 2000 bp/s), providing the largest foreign DNA (up to 170 kb) and protein (up to 1025 molecules) payload. Lambda phage has the ability to carry large nucleic acid sequences and the ability of capsid proteins to be modified by a variety of ligands make it an excellent gene and vaccine delivery vector. Its large capacity ensures phage vaccine can contain both cytokine genes and other adjuvant systems. In addition, it allows the insertion of genes with large introns, so it is very suitable for using as vaccines.

However, T7, T4 and lambda phages all need complex genetic engineering techniques to insert foreign genes into their genomes because of their large genomes. Among them, lambda phage has a mild lifestyle and may undergo lysogeny and lysis. This complex biological characteristic makes the titer of lambda phage lower than that of M13 phage. In contrast, MS2 phage has a simpler structure, making it more difficult to edit and engineer genes. However, its genome is short and can only carry relatively short fragments of foreign genes. Compared with other phages, MS2 as an RNA phage is very suitable for delivering RNA. In addition, MS2 capsids can undergo simple, site-selective dual surface modifications. Despite the high level of modification, the capsid remains assembled, making it a unique hybrid delivery vehicle. M13, T7, T4, lambda and MS2 as phage delivery vectors have their own unique advantages and disadvantages. Reasonable selection of phage delivery system suitable for specific application scenario can make more progress in the field of nanomedicine.

## Applications of phage-based delivery systems in disease therapy

In the fourth section, we have introduced the current engineering strategies of modifying phages to functionalize them. Through non-covalent modification, covalent modification, and gene modification strategies, phages themselves or combined with nanocarriers can be used as carriers for drug and gene delivery. Modified phages have broad application prospects in the field of nanomedicine. In this section, we discuss in more detail the application of phage-based delivery systems in disease therapy (Table 2), including antitumor therapy, antibacterial therapy and antiviral therapy.

**Table 2:** Summary of the cited reference for the use of phages themselves or in combination with nanocarriers as delivery vehicles for disease treatment

Modification method	Modification strategies [N]: Non-covalent [C]: Covalent [G]: Genetic	Species of phage	Modified material	Types of diseases	Refs.
Non-covalent modification	Electrostatic interaction	M13, S. typhimurium-specific phage	Silver nanoparticles, Cationic polymer PEI	Colorectal cancer, Bacterial infection	[36, 44]
Covalent modification	Bioorthogonal reaction	F. nucleatum-specific phage	DBCO-modified dextran nanoparticles	Colorectal cancer	[182]
	Amidation reaction	A. baumannii-specific phage, P. aeruginosa- specific phage, M13	NB, AIEgens, Pd nanozymes	Bacterial infection	[71, 187, 188]
	Gold-sulfur bonds formation	M13	Gold nanorods	Bacterial infection	[75]
	Nucleophilic substitution reaction	Sepsis pathogens-specific phage	AIE-PS	Sepsis	[189]
	Gold-sulfur bonds formation, amidation reaction	M13	Gold nanorods, Zn2^+^-binding peptides	Bacterial infection	[190]
Genetic modification	Phage display tumor-homing peptides and angiogenin-binding peptides, CEA-specifc scFv, VEGFR2, HCC-binding peptides, GM-CSF	fd388, M13, T4, MS2		Breast cancer, Colorectal cancer, Lung carcinoma, Hepatocarcinoma	[118, 175, 191–193]
	Phage display RGD peptides, delivery gene HSVtk, TNF-ɑ	M13-based AAVP		Brain carcinoma, Melanoma	[128, 130]
Hybrid modification	[N] Electrostatic interaction [G] Phage display negatively charged peptides, RGD peptides	M13	Cationic liposomes, PEI- modified microparticles	Breast cancer, Stroke	[43, 45]
	[N] Affinity interaction [G] phage display a gold- binding peptide and a prostate cancer cell-binding peptides	T7	Gold nanoparticles	Prostatic cancer	[52]
	[N] Hydrophobic interaction [C] Amidation reaction	M13	PCL-P2VP nanoparticles, folic acid	Nasopharyngeal cancer	[56]
	[C] Amidation reaction [G] Phage display SYPIPDT peptides, chlorotoxin peptides	M13	Rose Bengal, indocyanine green	Epidermal carcinoma, Brain carcinoma	[17, 194]
	[N] Affinity interaction [C] Schiff base reaction [G] Phage display glutamate sequences	M13	GA, PdNPs	Breast cancer	[54]

### Antitumor therapy

So far, there are few effective antineoplastic drugs because of the antitumor effect, high interstitial pressure and irregular tumor vascular system. More importantly, drugs may more effectively induce cancer cells to escape [177]. Traditional cancer treatments (such as surgery, chemotherapy) are often ineffective or inadequate, and even have serious side effects [178]. The development of nanomedicine makes it possible to use new cancer treatment strategies. It is crucial to choose an effective nanodelivery system and make drugs target tumor tissues to reduce toxicity and side effects of drugs [179]. Phage display technology and phage library screening methods have recently accelerated the pace of research, which has been widely used to find new tumor targeting molecules and deliver drugs through targeted strategies [180]. The displayed proteins or peptides can make the phage specific to some cancer-related cells and tissue components, and the drugs can enter the cells through endocytosis and inhibit or kill tumor cells. It also contributes to the accurate treatment of tumors with fewer side effects and avoiding the use of high doses of drugs [79]. Bar et al. [181] proposed that antineoplastic drugs, such as DOX, have combined with ErbB2-targeting phages and showed 1000-fold higher cytotoxicity against ErbB2-overexpressing cancer cells than free drugs. Therefore, the shortcomings of antitumor drugs may be overcome by preparing targeted drug-loading platforms.

**Fig. 7:**
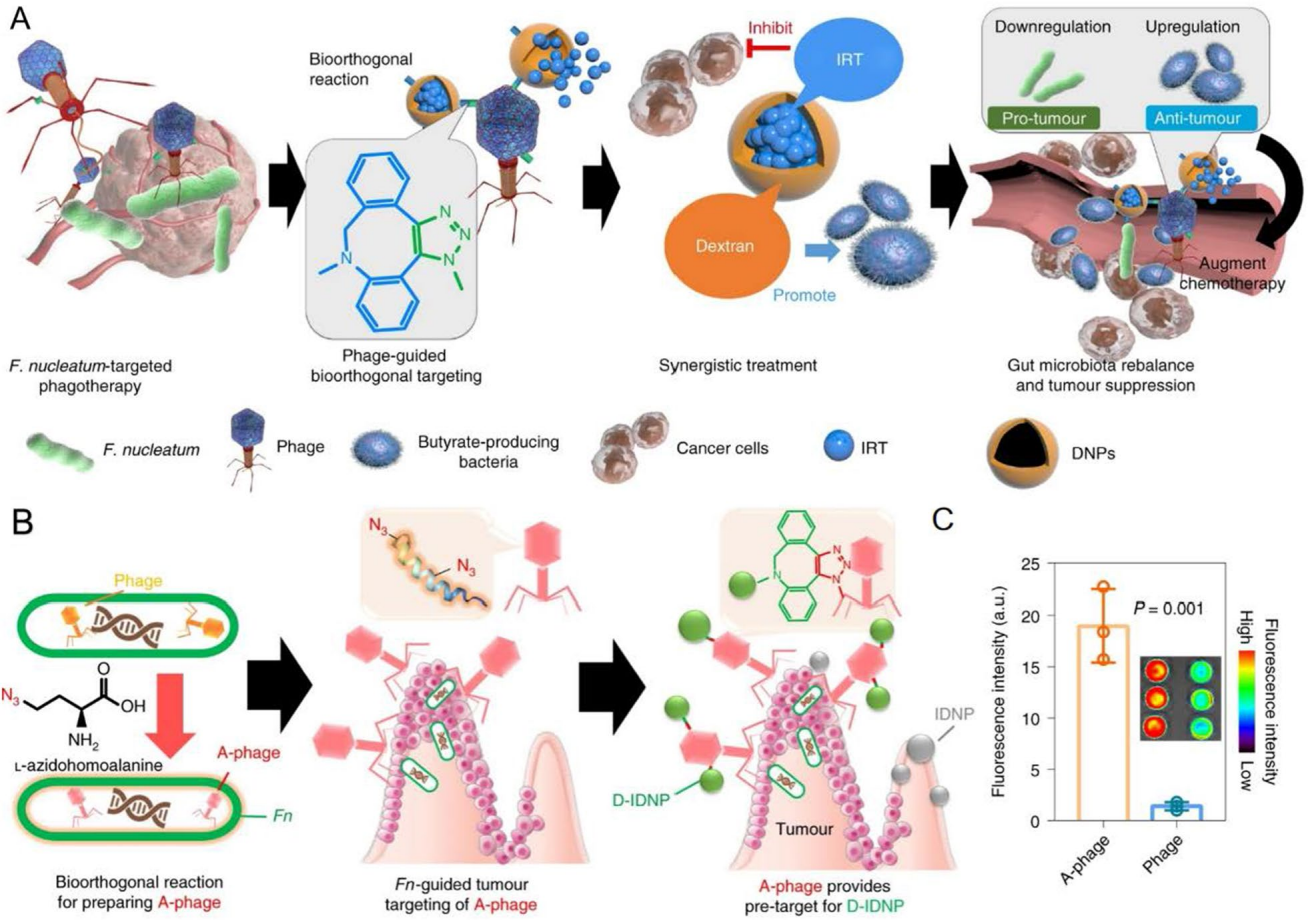
**A** Schematic representation of the phage-guided biotic-abiotic nanodrug delivery and its therapeutic effects. **B** Schematic diagram of tumor- targeting mechanism of A-phages (azide-modified phage) and D-IDNPs (azodibenzocyclooctyne (DBCO) modified irinotecan-loaded dextran nanoparticles). **C** Fluorescence imaging of covalent binding efficiency between A-phages and D-IDNPs. Reprinted from Ref. [182]

Phages attach to bacterial host cells through receptor-binding proteins (RBP) on virions, making them host-specific. If the therapeutic target is a bacterial species, RBP itself can be used for targeting [85]. At the same time, combined with other nanocarriers (such as nanoparticles), it is expected to further improve the therapeutic effect. Zheng et al. [182] developed a phage-mediated targeted nanomedicine. Using a bioorthogonal reaction, they covalently linked irinotecan-loaded dextran nanoparticles to azide-modified phage, and the nanomedicine inhibited the growth of *F. nucleatum* and significantly improved the first-line chemotherapy treatment of CRC efficiency (Fig. 7). Furthermore, the integration of phage display technology and nanotechnology is a potential method. The targeted peptides selected by phage screening have been successfully used as targeting parts to deliver drug nanoparticles to tumors. For example, Wang et al. [108] screened MCF-7 cell-specific peptides from a phage library. The peptide-fused phage pVIII protein was directly integrated into the liposome to successfully deliver the drug-loaded liposome to MCF-7 cells. This treatment modality reduces tumor volume, enhances antitumor activity and has no significant toxicity compared to non-targeted formulations [183]. The above studies demonstrated that targeted drug delivery mediated by phages combined with appropriate nanocarriers can enhance antitumor effects.

TME is characterized by abundant immunosuppressive cells as well as enriched immunosuppressive factors. This immunosuppressive network impairs the antitumor immune response [184]. Therefore, there is an urgent need to develop an effective immunomodulatory strategy to reverse immunosuppressive TME. As an emerging drug carrier, hydrogel can achieve sustained drug delivery at tumor sites and has been widely used in tumor drug delivery [185]. More recently, research groups have developed a bioactive gel system based on M13 phage. They constructed a self-adjuvant photothermal M13 phage gel coloaded NLG919 (M13@Pd/NLG gel) for PTT and immune synergistic therapy. Finally, the immunosuppressive TME was reversed and the anti-breast cancer response was improved [54]. Not only do hydrogels retain the structural integrity and functionality of their contents, they also provide additional engineered flexibility to enhance therapeutic efficacy [186]. In the future, through the reasonable design of phage-based gels, and combining the advantages of synthetic materials, the synergistic effect of them has the potential to improve the delivery efficiency of nanocarriers, thereby improving the therapeutic effect of tumors.

In conclusion, many therapeutic drugs, including anticancer drugs, antibiotics, cytokines, radionuclides, have been excluded from anticancer drug candidates because of their high toxicity and high dose [22]. So far evidence suggests that binding to tumor homing peptides can improve the treatment index of traditional chemotherapy drugs by reducing side effects and allowing higher drug accumulation at the disease site [21]. Therefore, the use of phage-mediated targeted drug delivery combined with nanocarriers can exert therapeutic efficacy and reduce toxic side effects. The strategy makes it possible to reintroduce drugs that have hitherto been excluded for cancer treatment. In the future, phage-based bioactive gel systems can be further developed to reverse the immunosuppressive TME and thus improve the antitumor effect. After all, hydrogels have excellent biocompatibility and biodegradability, and are less toxic than nanocarriers [185].

### Antibacterial therapy

Since the discovery of penicillin, antibiotics have been used primarily to treat bacterial infections. However, the global misuse or overuse of antibiotics has promoted bacterial evolution and exacerbated antibiotic resistance, posing a significant threat to human health [195, 196]. Phages, bacterial-specific viruses that can infect and inhibit host bacteria, have emerged as an important alternative option to combat antibiotic resistance in the current era of MDR pathogens [197].

Phages have been shown to be specific to their hosts and can evolve in parallel to adapt to infected drug-resistant bacteria. Thus, in antimicrobial battles, phages have been used to develop precise drugs to kill certain species of harmful pathogens, improving the targeting and killing efficiency of antimicrobial agents [57, 198]. However, the bacterial population at the infected site is usually a complex system, with not only multiple bacterial species but also a unique metabolic microenvironment, characterized by low pH, high reactive oxygen species (ROS), specific enzymes, and so on [199]. In order to overcome these limitations, Peng et al. [19] combined phages with gold nanorods to create chimeric phages. After engineering modification, they can specifically attach to several bacteria and melt them through photothermal ablation. Diseases infected by a variety of pathogens, such as sepsis, infection with the host blood system can lead not only to physiological disorders, but also to life-threatening organ dysfunction [200]. Phage cocktail therapy has been successfully used to save the lives of patients with sepsis in clinical practice. However, phages cannot be used in the diagnosis of sepsis due to the lack of imaging [189]. Fluorescence imaging is a fast, easy, and powerful tool for visualizing and tracking biological analytes, an indispensable technique for diagnosing disease. Therefore, the combination of photosensitizer and phage can achieve the purpose of real-time monitoring the therapeutic effect [187]. Wu et al. [189] proposed to combine aggregation-induced emission photosensitizer (AIE-PS) with phage through the nucleophilic substitution reaction between bromine and sulfhydryl groups for the treatment of sepsis. This synergistic strategy combining phage cocktail therapy and photodynamic therapy (PDT) exhibits strong bactericidal efficacy and provides a promising therapeutic platform for rapid pathogen detection and immediate diagnosis.

The utilization of phages holds great promise for addressing the worsening antibiotic resistance crisis, as the process of phage-induced bacterial lysis is not affected by bacterial resistance [188]. However, the short residence time of phages in the blood and their rapid clearance from the bloodstream limit their use [201]. Therefore, new innovative strategies to design long-circulating phages with antibacterial properties that prolong blood circulation time are highly needed. Jin et al. [202] identified a blood circulation prolonging peptide (BCP1) by screening the M13 phage display library in vivo, which significantly prolonged the blood retention time of human ferritin nanocages containing DOX. Subsequently, they extended the application of BCP1 to phage antimicrobial therapy by constructing M13 phage BCP1-BGL with long-circulating properties (by displaying BCP-1 peptide) and bactericidal activity (by expressing restriction enzyme BglII). The hybrid nanoparticles were further prepared through the physical combination of BCP1-BGL phage and PLT membrane, and showed significantly enhanced antibacterial effects [203]. This study provided an insight into enhancing phage therapy with nanoparticles based on engineering biomimetic phages.

In general, phages play an important role in the fight against antibiotic resistance in the current era of multiple drug resistance pathogens because of their high specificity, acceptable safety and efficacy in overcoming antibiotic resistance. In the future, further research is needed to prepare the next generation of antimicrobial agents to deal with antibiotic resistance.

### Antiviral therapy

The outbreak of COVID-19 has once again raised concerns about viral infection, which has posed a serious threat to human health throughout history [204]. So far, viruses that seriously threaten human health still lack eradication methods, such as human immunodeficiency virus (HIV) and hepatitis B virus (HBV). At the same time, traditional antiviral drugs have serious side effects, mainly causing mitochondrial DNA damage and hypersensitivity. Therefore, the development of effective antiviral strategies is of great significance for both known and possible emerging viruses [205].

Antiviral vaccines are the most successful biomedical interventions to protect individuals from viral diseases and prevent emerging viral infections [206]. It is recognized that phage display as a powerful technology represents great potential in a wide range of biomedical and pharmaceutical fields, including drug discovery and vaccine development [207]. At the same time, the CpG motif in the phage genome can produce a strong antigen-specific immune response through the TLR pathway, and the immune response to vaccine delivery can be enhanced by using an adjuvant-active phage [117]. Phages have been explored as antiviral vaccine delivery carriers for life-threatening infectious diseases. Phage-based vaccines can provide adequate protection against a variety of viral diseases, such as HIV [208], HBV [209] and herpes simplex virus type 1 (HSV-1) [210].

Phage display vaccines display foreign antigens as fusion proteins on the surface of phages. With phage display vaccines, recombinant phage displaying single or multiple antigens can be used to induce effective immune response and provide protection in viral infection [46]. This is especially true for some viruses, such as HIV, that have the ability to mutate to avoid immune responses. It is generally accepted that for an HIV vaccine to be effective, it should contain multiple antigens and generate strong and broadly neutralizing antibodies, as well as a cell-mediated immune response [211]. Sathaliyawala et al. [208] fused single and multiple antigens with Hoc capsid protein of T4 phage. HIV antigens displayed as many copies as possible on the surface of T4 phage, triggering powerful and broadly neutralizing antibodies in the absence of external adjuvants. Besides, filamentous phage is a universal platform for developing phage display vaccines. It was reported that Bahadir et al. [209] inserted hepatitis B core antigen (HBcAg) gene into the pIII of M13 phage. Then, mice were immunized with HBcAg-displaying recombinant phages, and their antigenicity and immunogenicity were tested to confirm the success of immunization. Recombinant filamentous phages can also be used as DNA vaccine delivery vehicles by engineering the phage genome to synthesize nucleic acid vaccines. Hashemi et al. [210] inserted the expression cassette of HSV-1 glycoprotein D into the M13 phage genome, and the constructed phage DNA vaccine could induce antiviral neutralizing antibodies and cellular responses in mice, thus showing that the phage vaccine was effective against HSV- 1 validity.

In addition to the long-term existence of viruses that pose a serious threat to human health, phage-based vaccines have great potential in preventing and treating emerging viral infections by triggering an effective immune response. It is impressive that the COVID-19 pandemic poses a serious threat to human life and health [204]. The phage has the characteristics of high stability, safety, low cost of mass production, easy gene manipulation and rapid identification of target protein, which makes it an ideal candidate for the development of COVID-19 vaccine [212]. For example, Staquicini et al. [213] reported two phage-based targeted vaccines against SARS-CoV-2. Dual ligand peptide-targeted phage was prepared by displaying the SARS-CoV-2 spike (S) protein and the peptide CAKSMGDIVC on pVIII and pIII, respectively. Targeted AAVP particles are designed to deliver the entire S protein gene under the control of a constitutive CMV promoter. Both triggered strong systemic and specific immune responses in mice without any evidence of adverse effects. Although SARS-CoV-2 is a rapidly evolving virus, and many variants of the virus have been observed during the COVID-19 pandemic [214]. The adaptability and flexibility of phage vaccines are potential advantages over emerging variants of SARS-CoV-2.

All of these studies have shown that phages as delivery vectors are promising against known and emerging viral infections. Phages are not pathogenic to humans, with negligible reports of infection, and have a potential adjuvant capacity to stimulate the eukaryotic immune system [215]. It is a unique nanoparticles platform that can be used to prepare multivalent vaccines against high-risk pathogens in order to quickly produce viable antibodies against any plague or pandemic in the future [216]. Currently, it is crucial to develop viral vaccines that can induce a long-term protective immune response without compromising safety and tolerance. Future research needs to optimize the phage vaccine platform to expand the diversity of phage vaccines to provide new strategies for the prevention and treatment of viral infection.

## Current status of phage application

Since Richard Bruynoghe and Joseph Maisin first reported in 1921 that the injection of phages into and around surgically opened lesions effectively treated staphylococcal skin disease, the mainstream clinical application of phages has focused on their conversion into "antibiotics" that can treat common resistant diseases. Treatment with medicinal bacteria has also achieved positive clinical expectations [217]. For the MDR bacteria that are common in clinical practice, the current phage preparations mainly focus on *Pseudomonas aeruginosa* (*P. aeruginosa*), *Acinetobacter baumannii* (*A. baumannii*), *Klebsiella pneumoniae* (*K. pneumoniae*), and *Staphylococcus aureus* (*S. aureus*) [218]. So far, no adverse immune reactions caused by the application of large doses of phages in vivo have been found [219]. Recent studies have shown that the use of phages for decolonisation is usually safe, considering the threat posed to humans by refractory bacteria and the importance of colonization in subsequent infections and pathogen transmission, the use of phages will be an effective alternative to bacterial decolonisation [220].

In addition, clinical applications related to phages include monoclonal antibodies derived from phage display. Today, monoclonal antibodies (mAbs) have become the dominant product category in the biopharmaceutical market [221]. In the early 1990s, John McCafferty and Sir. Gregory Winter developed an in vitro antibody selection technology called antibody phage display, which enabled the discovery of antibody-based drugs for a variety of purposes. Since then, more than 70 phage-derived antibodies have entered clinical studies. Currently, Food and Drug Administration (FDA) has approved 14 phage display-derived antibodies and antibody fragments. Monoclonal antibodies derived from these phage displays have made significant contributions to the diagnosis and treatment of diseases [222]. Unfortunately, as mentioned in the previous section, despite the promising applications of phages as delivery vehicles, so far there are no reports of human preclinical/clinical studies on phages as delivery vehicles. Although phages as nanomedicines have been widely used in animal experiments, there is a large gap between treatments in humans and animal experiments. Given that phage preparations have successfully treated human diseases (such as against MDR bacteria) without causing immune adverse reactions [136]. Therefore, it will be promising to clinically study the use of phages in the diagnosis and treatment of cancer and other challenging human diseases. Once the use of engineered phages as delivery vehicles to treat human diseases is demonstrated in clinical trials, it is likely that such engineered phages will receive clinical approval from FDA.

Although the clinical application of phages as delivery vehicles may take some time, it is believed that with the continuous deepening of research, the mechanism of phage action will continue to be revealed, and the regulation of phage therapy will continue to be determined, opening the door to the clinical application of phages.

## Summary and prospects

Phages are abundant and diverse [223]. The small size and variable shape of phages (filamentous and icosahedral) allow each type of phage to have its own characteristics and we can choose the appropriate carrier according to the purpose. Currently, the most popular and mature type of phage in cargo delivery is filamentous phage, more research needs to be carried out to further explore the functions of more types of phages in the future.

Among many different types of nanocarriers, phages have many advantages. Phages have high safety because they only specifically infect bacteria and have no infectivity to mammalian cells, which is a significant advantage compared to eukaryotic viral vectors [16]. There are a large number of functional sites on the surface of phages, which have high loading capacity. The amino acids in the capsid protein provide a variety of reactive functional groups that can be used for bioconjugation, and their physical and biological properties are optimized by drug conjugation [72], providing the possibility of reintroducing drugs that have been excluded as therapeutic agents so far. The application of phage display technology has opened up a new way to use phages to design targeted gene and drug delivery vectors. Various peptides and proteins can be successfully displayed on the capsid protein of phages. Phages can provide handles for chemical modification by displaying cell or tissue targeted peptides discovered through in vitro or in vivo biological selection, and allow attachment of parts for loading cargos [135]. Foreign genes can be inserted into the genome of phages through genetic engineering methods to improve the efficiency of gene transduction. Although phages are superior to other nanocarriers in some aspects, it is undeniable that other nanocarriers also have their unique advantages. The use of phages alone or in combination with other nanocarriers for drug and gene delivery may be the future solution, which is expected to improve the therapeutic effect.

Despite the interest in the study of phages as delivery systems, there are still the following limitations that need to be further overcome. Phages are an exogenous antigen with immunogenicity, which makes them perform well in vaccine applications. However, when using phages as therapeutic vectors continuously, it is necessary to consider the immunogenicity and retention time of phages [224]. Phages are often metabolized or cleared by the human body or immune system to reduce delivery efficiency, which requires special measures to avoid recognition and uptake [225]. At present, the extensive clinical application of phages is mainly focused on antibacterial therapy, and there are still safety issues that need to be addressed in the phage preparations used in clinical practice. In the future, with in-depth research on phages, the mechanism of phage action will continue to be revealed, and the existing problems are expected to be solved. With the continuous development of phage-based delivery systems, the application of phages will provide broad prospects for the prevention and treatment of various diseases, such as cancer and neurodegenerative diseases, ultimately opening the door to the clinical application of phages.

## Data Availability

The datasets used in this study are available from the corresponding author on reasonable request.
